# The conquest of the dark spaces: An experimental approach to lighting systems in Paleolithic caves

**DOI:** 10.1371/journal.pone.0250497

**Published:** 2021-06-16

**Authors:** Mª Ángeles Medina-Alcaide, Diego Garate, Iñaki Intxaurbe, José L. Sanchidrián, Olivia Rivero, Catherine Ferrier, Mª Dolores Mesa, Jaime Pereña, Iñaki Líbano

**Affiliations:** 1 Department of History, University of Córdoba (UCO), Córdoba, Spain; 2 International Institute for Prehistoric Research of Cantabria (IIIPC), University of Cantabria, Santander, Spain; 3 Department of Geology, University of Basque Country (UPV/EHU), Leioa, Spain; 4 Department of Prehistory, Ancient History and Archaeology, University of Salamanca (USAL), Salamanca, Spain; 5 UMR 5199, PACEA, University of Bordeaux, Bordeaux, Spain; 6 Research Institute Nerja Cave (IICN), Nerja, Spain; 7 Edestiaurre Arkeologia Elkartea, Barrika, Spain; University at Buffalo - The State University of New York, UNITED STATES

## Abstract

Artificial lighting was a crucial physical resource for expanding complex social and economic behavior in Paleolithic groups. Furthermore, the control of fire allowed the development of the first symbolic behavior in deep caves, around 176 ky BP. These activities would increase during the Upper Paleolithic, when lighting residues proliferated at these sites. The physical peculiarities of Paleolithic lighting resources are very poorly understood, although this is a key aspect for the study of human activity within caves and other dark contexts. In this work, we characterize the main Paleolithic lighting systems (e.g., wooden torches, portable fat lamps, and fireplaces) through empirical observations and experimental archeology in an endokarstic context. Furthermore, each lighting system’s characteristic combustion residues were identified to achieve a better identification for the archaeological record. The experiments are based on an exhaustive review of archaeological information about this topic. Besides, we apply the estimated luminous data of a Paleolithic cave with Paleolithic art (Atxurra in northern Spain) in 3D through GIS technology to delve into the archeologic implications of illumination in Paleolithic underground activities.

## 1. Introduction

Humans cannot see in the dark; therefore, they need light to enter the deep parts of caves, and their visits to those places depend on the physical characteristics of their lighting systems. The luminous intensity, radius of action, type of radiation, and color temperature of the light determine the perception of the environment and the human use inside (such as the execution of art, funerary activities, and cave exploration). The light duration restricts the time spent inside the cave and defines whether the visit will be a long stay or a short exploration. Moreover, the optimal management of some of the produced gases (i.e., the smoke of lighting tools) is essential to carry out prolonged subterranean frequentation [1–3].

This study aims to a better understanding of the lighting systems used in caves by Paleolithic societies through an empirical, experimental study at an endokarst site. The replicated and assessed lighting systems are based on the archaeological evidence found in the deep parts (named the “internal archaeological context”) of the Paleolithic caves in Southwest Europe. According to M Álvarez and D. Fiore [4], experimentation and the archaeological record must be connected by a dialectic link; research questions that arise from the archaeological evidence lead and determine the design of the experiments. And the results obtained through the latter are tools that provide a new type of knowledge about the former.

This internal archaeological context corresponds specifically to caves with Paleolithic art. This is because these types of studies for this period have been mainly directed toward decorated caverns. There have been very few studies concerning caves’ internal archaeological context without Paleolithic graphic activity [5]. We will focus on the deep parts of caves to track the Paleolithic lighting systems because we can ensure in those sites that the fire had a functional purpose related to lighting without excluding other additional types of activities.

A better understanding of the Upper Paleolithic lighting used to access and utilize the deep parts of caves will enable a more precise comprehension of the activities carried out, which are closely linked to the origins of human symbolic and artistic behavior. This study has quantitatively characterized, for the first time, the main luminosity aspects of Paleolithic lighting systems based on archaeological and empirical data. This information is of great interest to the scientific community. It is essential for the sensorial analysis of frequented deep spaces in Paleolithic caves through different technological solutions (geographical information systems and three-dimensional replications, among others), including the realistic dissemination of the cultural heritage located in those sites.

### 1.1. Archaeological support: Remains related to lighting in Paleolithic caves

The first solid data about the presence of ancient humans in the inner parts of the caves (in places with total darkness, where artificial lighting is indispensable) was linked to Neanderthals, with evidence in Bruniquel Cave (France), where six anthropic circular structures formed by 400 fractured speleothems 336 meters from the entrance contained more than 18 traces of fire (probably burnt bones). A time of 176 ky BP has been proposed for this activity by uranium-series dating of calcite stalagmites, particularly the tops of stalagmites that are part of the structure (maximum ages) and of the bases of the stalagmite regrowth that seal the structures (minimum ages) [6]. There have been other proposals regarding human activity in the deep parts of caves, such as the deposit of human remains approximately 400 ky BP in Sima de Los Huesos in Atapuerca (Spain) [7], Dinaledi Chamber (South Africa) in 300–200 ky BP [8], Lamalunga Cave (Italy) in approximately 172–130 ky [9], or Vârtop Cave (Romania) in 60 ky [10]. Nevertheless, without considering the intentional origin of those remains, neither evidence of the use of fire, as in Bruniquel Cave, has been found at any Upper Paleolithic site, nor archaeological remains related to lighting proliferation has been found, especially remains associated with human uses in deep endokarst contexts linked to Paleolithic art. In those dark sites, combustion traces of three Paleolithic lighting systems have been recognized, particularly torches, fireplaces, and portable grease lamps [11, 12].

#### 1.1.1. Torches

Three peculiarities characterize the residues of torches: a) their dispersal location on the ground in caves, created by the intermittent fall of combustion residues (usually wood charcoals) along paths due to their use (such as the breadcrumbs in the story of *Hansel and Gretel*). b) Their location in the deep parts of the caves, where the artificial lighting is indispensable. We can be sure that the fire resources were used for artificial lighting in these places without discarding other additional activities (such as space markers, refueling sites, or with more cultural or symbolic meaning ones). c) The confirmation of its location in primary position (discarding those combustion remains coming from other contexts outside of the cave).

**Fig 1:**
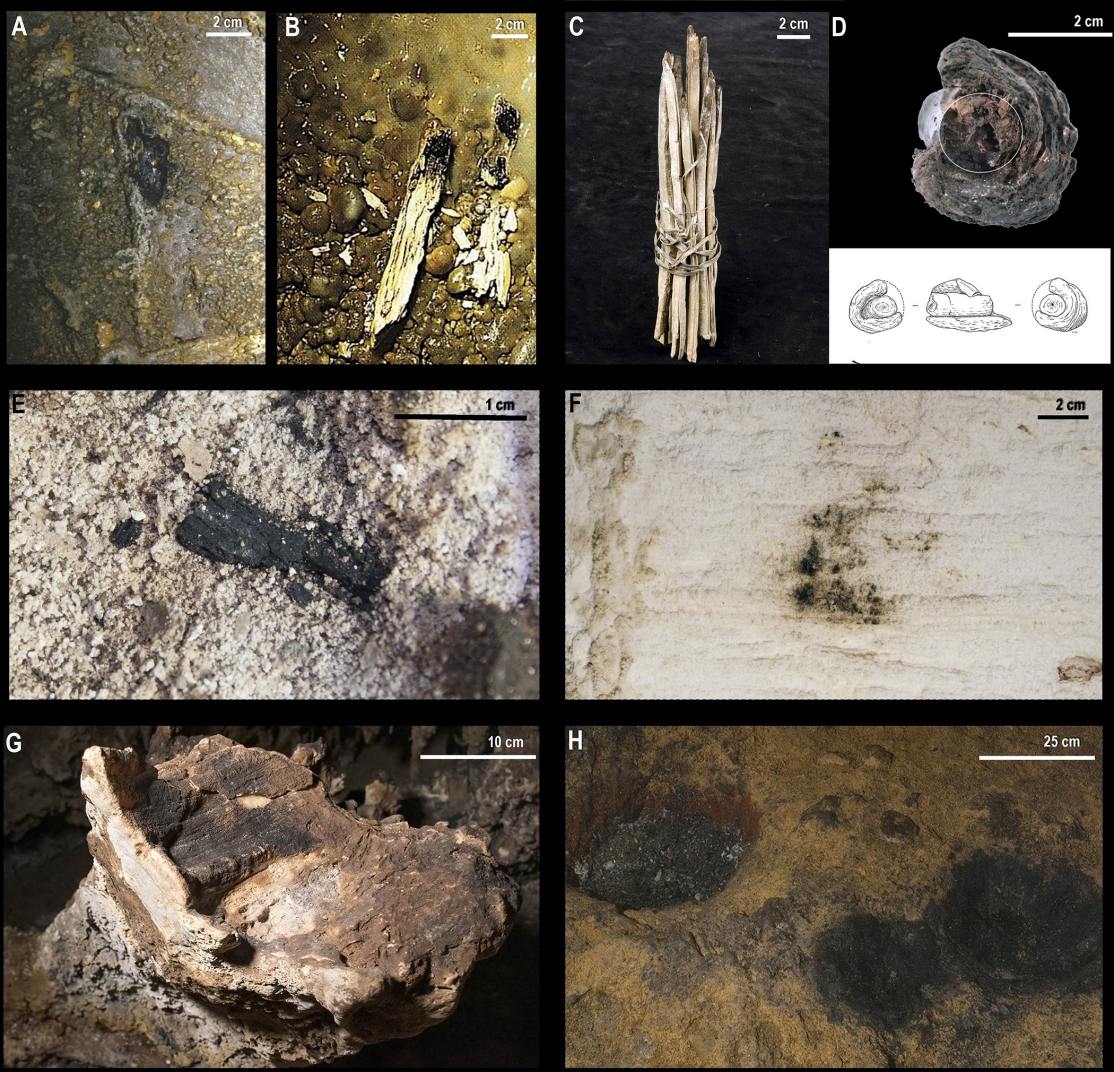
Prehistoric lighting remains. A. Fragments of semi-charred juniper branches covered by calcite in the cave of Aldène. Republished from ref. [16] under a CC BY license, with permission from Philippe Galant. B. Portion of pine wood in the Réseau Clastres Republished from ref. [14] under a CC BY license, with permission from Jean Clottes. C. Torch preserved in the mines at Hallstatt. Published under a CC BY license, with permission from Markus Roboch, CFO in Natural History Museum Vienna. D. Side view and illustration of the Star Carr torch Published under a CC BY license in ref. [17]. E. Scarred charcoal from Nerja Cave; F. Black marks from Nerja Cave; G. Fixed lamp from Nerja Cave. H. Fireplaces in a pit in Enlène Cave. Published under a CC BY license, with permission from Jean Clottes].

The residues of torches of Paleolithic age found in the caves’ inner parts are usually limited to scattered charcoals (Fig 1E) above different surfaces, and black marks on the walls and ceilings (Fig 1F).

Additionally, although they do not belong strictly to Pleistocene chronologies, there are some examples related to other hunter-gatherer societies that frequented the same caves as Paleolithic art.

For example, there is an outstanding vestige of a torch of Epipaleolithic age in the cave with Paleolithic art at Réseau Clastres (Ariège, France) that is linked with some kind of visits after Paleolithic graphic activity ([13, 14]: Fig 1B). This is an 11 cm long fragment, partially charred and covered with calcite, with a flattened cross-section. Furthermore, in this cave, there has been an enormous assemblage of scattered charcoals and black marks. The anthracological study of these charcoals determined the almost exclusive use of *Pinus sylvestris* (scots pine) for lighting in at least four different visits between 4.5–10.1 ky BP [13].

Other unusual vestiges of torches were found in the “Footprints Gallery” of the cave with Paleolithic art at Aldène (Hérault, France) [15, 16]. Again, there were dispersed pieces of charcoal and black marks on the walls also linked to the use of torches during the Epipaleolithic period (between 8.9–7.8 ky BP). Additionally, there are some small partially charred branches under a layer of calcite (Fig 1A). These remains are very useful for appreciating the thickness of the branches used for lighting. These vary between 1 and 2 cm wide and up to 27 cm long, although most of them measure between 10 and 20 cm in length.

Finally, other remains of torches found in other contexts but with prehistoric chronologies have helped conceive their potential morphology (for example, the outstanding example of Star Carr in the United Kingdom) (Fig 1D). Star Carr is an open-air Mesolithic site located in a peat bog on a riverbank. Over 200 rolls of semi-charred *Betula* sp. (birch) bark have been identified there, of which two were interpreted as torches. These two are of birch branch construction with the same species’ bark rolled around their distal end [17]. Moreover, numerous remains of torches used in salt mining have been preserved in Bronze and Iron Age mines at Hallstatt (Upper Austria, Austria). The wood generally came from fir in these locations and was cut into thin longitudinal sections up to 5 cm wide (Fig 1C) [18].

#### 1.1.2. Fireplaces

On the other hand, the remains of fireplaces related to lighting, like the remains of torches, are defined by their location in the deep and dark areas of caves, where their use for lighting is unquestionable (without discarding other additional possible functions such as space markers, refueling sites, or with more cultural or symbolic meaning ones). In these cases, the combustion residues are concentrated instead of scattered (as is the case for torches) and can include different types of remains: charcoal, burned bones, ashes, rubefaction, or soot. In Nerja Cave (Andalusia, Spain), for example, some residues of fire have been found in a natural concavity on a speleothem (Fig 1G). A multi-analytical methodology has characterized this fireplace and its remains of ashes, charcoals, and soot, dated by C14-AMS between 22,500–22,200 years cal BP, in the Solutrean period [19]. In Enlène Cave (Ariège, France), fireplaces inside clay hollows excavated in the floor in the cave’s deep parts have been found (Fig 1H). They have been attributed to the Magdalenian approximately 15,000 years cal BP [20], and they contain burnt bones and show the presence of rubefaction. Likewise, large fires have been documented in Chauvet Cave by a taphonomic study of heated walls [21, 22] and related through thermoluminescence analysis with the first phase of graphic decoration in the cave (37 to 33,500 years cal. BP) [23].

#### 1.1.3. Portable grease lamps

The use of portable stone lamps fueled with animal fat has been attested to by the location of hundreds of these pieces (or fragments of them) in contexts mainly attributed to the Magdalenian [11]. These lamps constitute a varied record and appear in different forms. Some of them were carefully prepared (a minority), others had only been altered slightly [24], and some flat or concave stones were used without any preparation [11, 25]. They functioned as open or closed circuits [11]. Most of the lamps employed a stone object (such as limestone, granite, or slate), but also shells were used [26], and it has been proposed that bone and vegetal objects were utilized [27]. Besides, other static lamps with higher volumes have been found (e.g., ref. [28]). In sum, numerous forms of Paleolithic lamps existed, but they all must fulfill the criteria to be defined as lamps: the scientific determination of remains or residues of combustion left by their use. This is the only proof that can differentiate lamps from other objects with a similar morphology (e.g., palettes to mix colorants) [11] or a simple natural formation (e.g., natural geodes). A suggestive appearance and shape, generally concave, suitable for use as a lamp, together with blackish or reddish marks on some part of them, were often sufficient for classification as a lamp in previous works. Indeed, numerous objects are described as lamps without any serious study to characterize them.

The combustion remains in Paleolithic lamps may take different forms: the carbonized remains of the wick; signs of smoke, soot, or rubefaction on parts of the lamp; or animal fat used as fuel preserved on it [29]. Until now, there have been very few examples of verification of combustion residues on Paleolithic lamps. The analysis of S.A. de Beaune is an exception in this issue [11]. Here, of the 302 objects examined might have been used as lamp, only 85 could definitely identified as such.

### 1.2. Fuel used in Paleolithic lighting systems

Wood is the fuel that has been mostly registered in Paleolithic caves linked to the use of torches and fireplaces for lighting. A more occasional use of bone fuel has also been detected as well at the Enlène (Ariège, France) [20] and Alkerdi 2 (Navarre, Spain) caves [30]. The combustible properties of wood have been thoroughly characterized in previous studies [31, 32]. In this sense, we have to consider that not all animal and vegetable fuel remains have the same features for preservation (i.e., soft tissue remains are very perishable). We must also consider the difficulty of preserving the elements that have not been charred.

**Fig 2:**
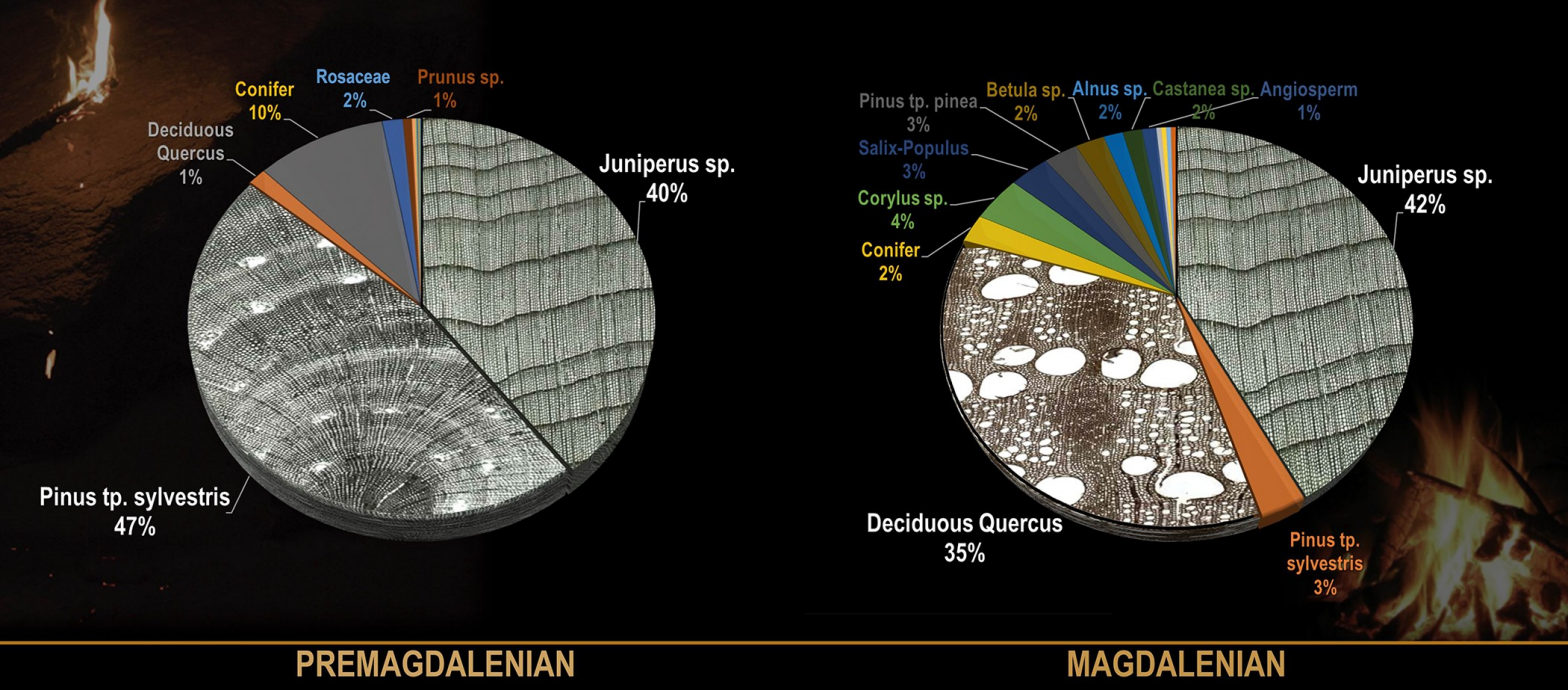
Anthracological data from Paleolithic light systems (torches and fireplaces). For more information: *S1 Appendix*. Wood transversal section images: www.woodanatomy.ch.

However, numerous charcoals have been found in decorated caves’ internal areas, even if they have not been the main subject of prior studies [5]. Table 1 (*S1 Appendix*) collects the scarce anthracological data from exclusive upper Paleolithic contexts found in the deep parts of caves, separated from the outside of the habitat sites in their entrances, together with information about the chronology of the visit with which they are associated. These data have been summarized in Fig 2. For the pre-Magdalenian period, there was a preferential selection of wood from *Pinus* tp. *sylvestris* (compatible with the woods of scots pine, black pine and mountain pine) and *Juniperus* sp. (juniper), in addition to other taxa equal to or less than 2%. For example, in the previously mentioned fireplace from Nerja cave, with a Solutrean chronology (22.5–22.2 ky Cal BP), the exclusive use of *Pinus* tp. *sylvestris* as woody fuel has been identified [19]. For the Magdalenian period, the use of *Juniperus sp*. remains, considerably reducing *Pinus* tp. *sylvestris’* use, and appearing as an important selection of deciduous *Quercus*, together with other taxa equal to or less than 4%. This reflects a remarkable selection of fuel for underground lighting activities, which has been previously pointed out by other researchers [33–35]. This idea is reinforced if we analyze each site in particular; in fact, most of the internal sites with available anthracological data have only 1 or 2 different species (*S1 Appendix*).

**Table 1:** Average values of the measured parameters in each experiment.

LIGHTING TYPE	TORCHES						LAMPS			FIREPLACE
*Number of experiment*	**1**	**2**	**3**	**4**	**5**	$\bar{x}$	**6**	**7**	$\bar{x}$	**8**
**Max. duration (’)**	31	19	44	50	61	41	>60	>60	>60	>30
**Average E (lux)**	19.08	12	9,98	21.61	21.94	16.92	2.97	3.71	3.34	19.2
**Average I (cd)**	3.05	1.92	1.60	3.46	3.51	2.71	0.47	0.59	0.53	3.07
**Average Radius (m.)**	2.93	3.20	3.60	2.73	2.47	2.99	1.52	1.57	1.54	3.30
**Average L (cd/m**^**2**^**)**	2.43	1.53	1.27	2.75	2.79	2.16	0.38	0.47	0.43	2.45
**Average High Temperature. Center (°C)**	537.80	307.25	662.00	666.25	633.88	561.44	144	176.33	160.16	586.67
**Average High Temperature. Periphery (°C)**	128.60	35.00	58.20	63.88	277.50	112.64	-	-	-	45.00

Some researchers have debated this selection by Paleolithic groups with various explanations. This could be conditioned by environmental restrictions, functional purposes (such as the suitability of these woods for lighting, as could be the case for resinous woods), or other cultural aspects related to wood management. However, the first option seems to be ruled out in most cases based on other paleobotanical data that do not reflect the exclusivity of these woods close to the caves [33–35].

We must make two points regarding the choice of woody fuel for frequentation and lighting in underground contexts. First, anthracological studies are still limited, and they are excessively focused on taxonomic analysis; thus, they do not allow general conclusions about the choice of woody fuel yet. Second, a strong selection of woody fuel is also reflected in external Paleolithic sites and does not appear exclusive to internal sites. In short-term structures such as hearths, a low representation of species is particularly frequent [36–39]. Furthermore, fire mono-functionality has been related to a strong selection of fuel based on ethnographic data [40, 41].

Lastly, there are some data on the fuels used for portable grease lamps (mainly from anthracological and chemical analyses). Regarding wood used for wicks, juniper has been identified in the best-known lamp from Lascaux Cave (Dordogne, France), although the possibility that it is *Taxus baccata* (yew) cannot be ruled out [24]. Carbonaceous samples from calcareous plaquettes at Lascaux have also been identified: nine were described as an amorphous mass other than wood, two as charcoal from an indeterminate wood, one as a *Gramineae* (grass) twig, seven as conifer charcoal, four as juniper charcoal, and one as bone [11, 24].

Other substances were detected by microscopic observation of the lamps from Lascaux Cave, such as wood charcoal ash and a possible amorphous substance related to resin (perhaps from the combustion of wood from a resinous conifer). The latter determination was made from the residue on the famous ‘lamp’ made of pink sandstone in the cave [42]. The presence of carbonized osseous remains has also been proposed for the ‘fixed’ lamps in Ardales Cave (Andalusia, Spain), without ruling out other possible sources for phosphates’ remains, such as the rock itself or bat excrement [43].

**Fig 3:**
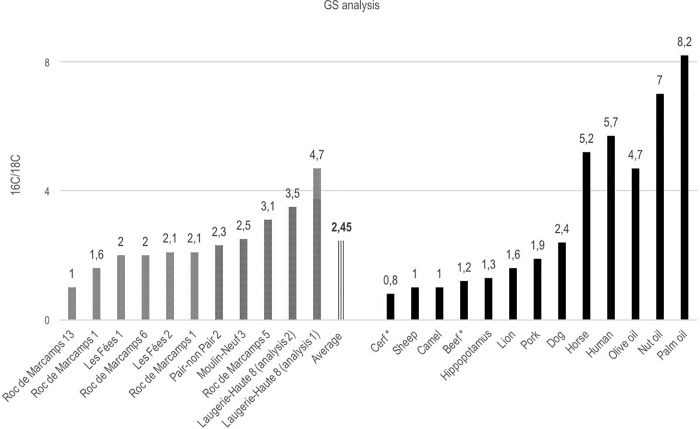
GS-analysis data. Paleolithic lamp (left) and a reference index of vegetable and animal fat (right) (modified from ref. 11: 40). The fat from animals normally consumed in the upper Paleolithic is marked with an asterisk.

Several gas chromatological studies have been carried out on the combustion residue from fatty fuel on Paleolithic lamps [11] (Fig 3). The mean 16C/18C ratios obtained for the archaeological samples can be compared most closely with the animal fat from bovids or deer if other animal species rarely found in Paleolithic deposits are excluded.

### 1.3. *Looking at the past*: Previous experimental studies

Experimental studies are essential for approaching the potential of Paleolithic groups for lighting used in caves. However, scientific experimental approaches to the topic are scarce, and most studies have focused on one type of light source: portable stone lamps.

At the beginning of the 20th century, the first references about stone lamps were all related to the use of animal fat as fuel, following the results of the pioneering chemical analysis performed for the La Mouthe lamp [44]. In the second half of the 20th century, more detailed works on Paleolithic lighting in the context of the scientific consolidation of experimental archaeology [11, 24, 45] obtained the first quantitative data about lamps. B. and G. Delluc [24] designated a reflection coefficient, at 1 meter from the wall, of 40–50% of the light received and a color temperature of less than 3,000 K for the light emitted by their experimental lamp. M. H. Newcomer [45] indicated that 80 grams of lamb fat provided light for 5 hours with a single wick. However, the most complete data on this topic were published by S.A. de Beaune [11], who compiled an exhaustive bibliography together with a methodical experimental protocol based on archaeological data and ethnographical information from the Inuit. This research indicates that the luminance emitted by a Paleolithic lamp, measured at 1 m from the flame, is 0.12–0.15 lux.

The experimentation undertaken by A. Rigaud [28] to determine how black residues on La Garenne (Indre, France) lamps were produced provided alternative information to the usual mechanism followed for lighting Paleolithic lamps. Similar traces appeared when charcoal and burned spongy bone were used as wicks. These materials provided rapid ignition and large flames. On the other hand, E. Pérez and D. Muñoz [46] compared the potential of different types of natural fuels in terms of duration, illuminance, and flame temperature. Marrow and beeswax were the most balanced fuels concerning these parameters. In turn, resin provided great lighting and caloric potential, although the gases that stimulated these characteristics quickly volatilized. D. Ruiz-González *et al*. [47] published experimentation about bone marrow’s particular potential for lighting. It provided long durability and a stable flame, with an absence of smoke. To finish, L. Pitchford [48] has given an interesting lecture where she has evaluated the light intensity of paleolithic lamps using different porous wicks and animal fats.

Experimental work on fireplaces deep inside caves is limited. C. Ferrier and the CarMoThap team carried out the main research. These researchers detected through thermoluminic analysis of the heated walls and an experimental approach that the temperatures reached by the walls of Chauvet Cave (Ardèche, France), adjacent to the fires, were very high (> 300–375°C). In this sense, they propose that the purpose of these big fires could be related to functions additional to lighting, such as sanitation of the cavern, human protection from animals, smoke production without a direct practical need except in relation to the scenography of the decorated space, together with the voluntary modification of the coloration and consistency of the wall concerning the execution of Paleolithic art [21, 22, 49]. Likewise, they have developed some monitored experimental activities and numerical simulations of fires in caves [2, 3]. These results have been applied to the 3D geometry of Chauvet cave to better understand the functionality of these fires and Paleolithic underground activity [49]. In this cave, at least 10 fires were made by Paleolithic societies, using a total of 170 kg of fuel. The smoke dispersion would allow the fires to be replaced given the thermal stratification of the gases [2], and the hearths would be arranged in a tepee shape nearby but slightly away from the wall to accomplish this [49]. These fires’ high radiation would be the main problem for human occupation when they were alight [3].

Regarding experimental studies on Paleolithic torches, there are only a few specific references. G. Malvesin-Fabre and H. Parriaud [50]—thanks to a series of briefly described experiences—point out that juniper wood is more favorable than hardwood in terms of duration, intensity, and absence of smoke. They also indicate that pine provides very bright light, although it emits black smoke. F. Rouzaud [51] provided further data on the use of torches in the underground landscape, such as their suitability for transit in wide and high galleries, the ease of handling them, and their multidirectional radiation. He also indicated that lamps are not appropriate for more difficult passages due to the possibility of fuel spillage, although their operation is more durable and regular. In this sense, he points out that both lighting systems would be complementary in the human use of caves. Empirical data in our own experiments confirmed many of the observations proposed by this researcher. Moreover, we found out an experiment with torches carried out by C. Andrieux in the Niaux cave. Six scots pine torches, beeswax impregnated with 80 cm long and 1kg each were made. He could move about 4 kms in 3h with them inside the cave. He stated that despite being a smoking instrument, it is appropriate for transit through the cave and that it was not necessary to reactive them [52].

Galant *et al*. [16] provided the most comprehensive treatment of Paleolithic torches. These authors performed a test with experimental torches based on the morphology and arrangement of parietal black marks and the results of the anthracological analysis of pieces of charcoal from Aldène Cave (all identified as juniper wood). Specifically, they reproduced juniper wood torches that measured 70 by 10 centimeters in size. These torches provided light for 30–45 minutes within an average action diameter of 4–6 meters. The authors also make some interesting observations about the use of those torches in the underground environment. For example, they generated the largest amount of charcoal during the first minutes of lighting, and they could be oxygenated by moving them in the air. This lighting system allowed comfortable transit in caves, even in difficult sites, without dazzling. However, this work lacked quantitative measurements regarding the light’s intensity.

H. Moyes [53] carried out further experimentation on prehistoric torches based on archaeological, ethnohistorical, and iconographic data about the torches used by Mayan societies in ritual cave contexts. The torches were made with pine wood and were 50–70 cm long and 1–5 cm thick. These produced 22 minutes of flame and approximately 60 grams of charcoal residue spread over a fixed surface of 2 square meters.

Concerning the choice of fuel for lighting purposes, I. Théry-Parisot and S. Thiébault [33] demonstrated, in laboratory experiments, that the flame height of *Pinus* sylvestris wood is greater than that of *Quercus pubescens* (pubescent oak) and that the pine produces higher and more durable flames (due to its resin content). Furthermore, S. Hoare [54] identified, from an experimental study of hearths in outdoor contexts, that there are significant differences in light intensity and durability between different wood species and fresh bone. This researcher observes that the fuel types that maintain brighter and more durable light are *Fagus* sp. (beech), *Larix* sp. (larch), *Fraxinus* sp. (ash), *Pinus* sp. (pine), and fresh bone. In contrast, *Alnus* sp. (alder) and *Quercus* sp. (oak) produce a particularly short light duration. Likewise, several experimental approaches have pointed out the benefits of animal fuel (especially fat and spongy bone tissue) to extend combustion duration [28, 55–60].

### 1.4. Human vision

It is also necessary to bear in mind that certain characteristics of human vision influence light perception and, consequently, the appreciation of colors in a cave. First, lighted objects or those emitting light can be perceived for as long as they are within the human visual field: 130° vertically and 180° horizontally. Second, cones and rods are the photoreceptor cells in the eye responsible for capturing and processing ambient light’s different intensities. The first is located in the fovea of the eye and is very sensitive to colors. The second is located outside the fovea and is very sensitive to light and movement but not sensitive to color. In dark areas, with a luminous intensity between 0.01 and 0.003 cd/m^2^, the rods are primarily involved, enabling scotopic vision. Both photoreceptor cells are active under adaptation conditions at levels between 10 and 0.003 cd/m2 (mesopic vision). Finally, between 3 and 10 cd/m^2^ (photopic vision), the cones are at their maximum efficiency, and a defined perception of colors is possible [61]. Therefore, in low light areas, visual perception is less related to color and more related to the contrast between lit and unlit areas.

Furthermore, as luminosity decreases, the human retina loses sensitivity to short wavelengths (corresponding to green, blue, and purple) first, and then to long wavelengths (corresponding to yellow, orange, and red). Thus, red is best seen in low light conditions, considering that the color threshold for human vision is 3 lux [62, 63]. The perception of colors also depends on the color temperature of the light; under incandescent or warm light (between 1000 and 2000 K), such as firelight, a yellow hue is emitted, and colors tend to appear more vivid [64, 65].

Visual capacity depends not only on the physical parameters of the illumination but also on the shape and transparency of the different elements that make up a person’s optical system, the alignment, visual convergence, spectral sensitivity of the retina, and the age of each individual. For example, older adults have poorer vision, even though their eyes are typically more sensitive to glare. Simultaneously, the standard adaptation to darkness by humans takes between 30 and 45 minutes when passing from a lighter to a darker place, with an average total adaptation time of 30 minutes. From dark to light, the reverse takes just seconds, with the pupil decreasing from 8 millimeters (poor light conditions) to 2 millimeters in conditions with optimal lighting [61]. Lastly, insufficient light can lead to fatigue, reduced reaction capacity, and relative myopia of 0.5–1.5 dioptres [62, 66].

## 2. Materials: Experimental materials

### 2.1. Selected raw material

The replicated light resources are based as much as possible on archaeological data. In particular, dry, small caliber juniper wood was used to make the torches and the lamp wicks. As explained above, this type of wood has been frequently recorded in relation to light activities in Paleolithic caves, both for the Magdalenian and pre-Magdalenian periods. For the experiment with a small fireplace, several oak branches were used together with the juniper. These two types of wood have also been identified together in deep sectors of caves associated with fireplaces. In particular, we replicated the composition of those hearths found in Sector J of Atxurra Cave (Basque Country, Spain), where three fireplaces were laid out on the ground under a large composition of engraved and painted animals with Magdalenian chronology beneath a cornice [67].

For non-woody fuel, animal fats were used in some experiments for torches and portable lamps; this was bone marrow from a bovid and a deer, similar to the known data according to chromatographic analyses made of Paleolithic lamps [11]. Solid pine resin was also employed. The use of resin was identified in the case of the stone lamp from Lascaux, although without definitive confirmation [24]. However, most of the charcoal linked to lighting in caves with Paleolithic art comes from resinous wood, especially *Pinus* tp. *sylvestris*-*nigra* and *Juniperus sp*. (Fig 2) Therefore, we consider that its use can be indirectly attested to.

Overall, green ivy and dry birch bark have been used to make the torches. These materials have not been identified at the moment in the examined contexts, perhaps due to preservation issues, but their availability has been characterized in other sites with Paleolithic chronology [68]. In any case, they are useful materials for making torches, as evidenced by the remains found in favorable contexts for preservation but with more recent chronologies [17, 18]. Specifically, ivy has been used as a cord to unite the different elements that make up the torches, and any other string-like element could replace this. Birch bark has been used to light fires due to its high flammability and rapid combustion.

**Fig 4:**
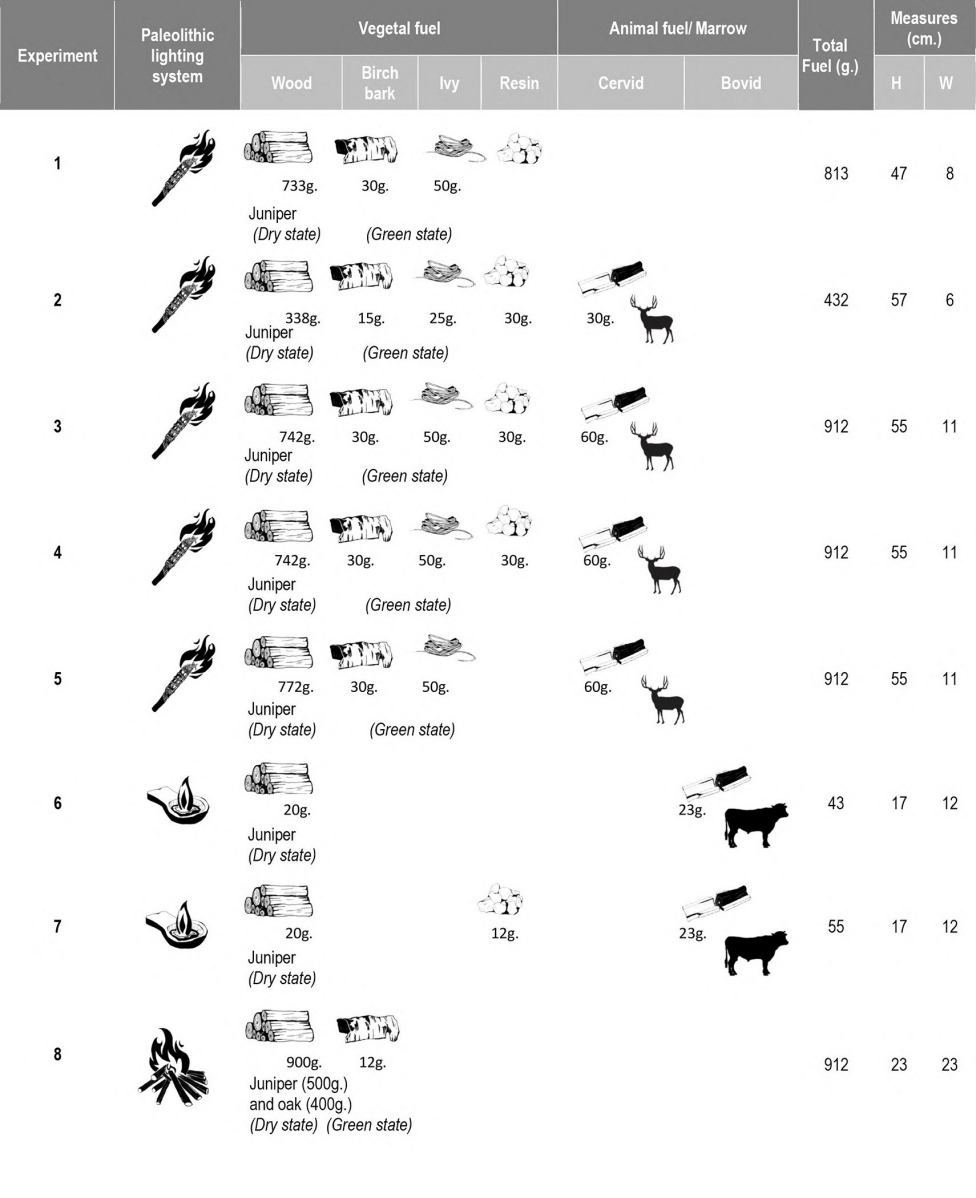
Fuel composition and measurements of the replicated torches, lamps, and fireplaces.

In **Fig 4**, we synthesize the raw materials used in each experiment (more precise information about the fuel used in the experimental activities is detailed in *S4 Appendix*).

### 2.2. Reproduced lighting types

Three different types of Paleolithic lighting sources were assessed in eight experiments: five torches, two stone lamps with animal fat, and a small fireplace. Their measurements (size and weight) are summarized in Table 1. More detailed information about the materials in each experiment and their preparation is given in *S4 Appendix*.

Torches: The functioning of five wooden torches has been evaluated in a cave context (experiments 1–5). They were all made from branches of dry juniper wood 1.2 cm thick that were joined together. This type of structure was chosen because it fits with the archaeological data and the form of the few prehistoric torches that have been preserved (see Section 2.1). For example, this matches the thickness of the remains of branches that have been found in the caves of Aldène and Réseau Clastres and with the morphology of a Hallstatt torch. Birch bark was included among the juniper branches as a kind of tinder to start combustion. Similarly, the wood was broken up to facilitate combustion (through easier oxygenation) and impregnation with non-ligneous fuel. Thus, pine resin, animal fat, or a combination were added in some cases to assess the function of the torches with those fuel types. The torch experiments were carried out and evaluated in zones A and B of the experimental cave (*S2 Appendix*).

Lamps: The lamps used in the experiments were replicas of the lamp from La Mouthe Cave (Dordogne, France) [44]. This lamp was made in sandstone, 17 cm long and 12 cm wide, and had a concavity with ≈150 cm^3^ capacity. Bovid marrow was used as the main fuel, with three woody wicks comprised of dried and crushed juniper wood. They were arranged in a tepee shape in the center of the active part of the piece. Pine resin was added in one experiment (number 7) to assess this vegetal fuel’s benefits for light. Lamp experiments were performed and evaluated in Zone A of the experimental cave.

Fireplace: A small fireplace was examined through an experimental study (experiment 8). The wood fuel was thin branches of juniper and oak wood in a dry state, arranged in a tepee-shaped structure. Birch bark was used to start the fire. This was placed inside the combustion structure. The fireplace was 23 cm in diameter and 7 cm long from the ground (measured before lighting), had no boundary structure, and was lit on a clay substrate. This experiment was also carried out in Zone A of the experimental cave at a distance of 80 cm from the closest wall and 1.60 m from the ceiling.

### 2.3. Endokarstic environment

The combustion process is influenced by environmental temperatures and humidity [69]. For this reason, experimentation was carried out inside a natural cave after thoroughly inspecting the area to ensure that there were no archaeological remains. In addition, this allowed us to evaluate lighting systems in direct relation to frequency and subterranean transit [70]. To be precise, the experimentation took place at Isuntza I Cave (Basque Country, Spain). This was formed in massive coralline limestone as a part of the Early Cretaceous Urgonian limestone complex. The cave’s most open sectors were selected to avoid light reflectance on the walls, ceilings, and other endokarstic obstacles as much as possible.

The experimental activities took place in two distinct spaces (*zones A and B, S3 Appendix*). The first is a wide chamber, 12.6 by 6 m in size and 7 m long. The floor is horizontal, consisting of rock, flowstones, and some dry clay, and the walls are made of bedrock with sparse lithochemical formations. The average relative humidity in this part of the cave is 99.7%, and the average temperature is 17.6°C. This chamber could be considered a “staying area” [71] since the horizontal floor and the large dimensions would allow a comfortable stay. This is where a small fireplace was built to light the torches. Subsequently, the torches were measured while walking along a large passage (4.5 m wide by 5 m long) approximately 40 meters long, which could be considered a “regular transit area”. They were also placed in a smaller chamber (4 m wide by 2 m long) and their parameters were measured.

The second zone is a wide area (9.4 by 9 m) and 4.5 m high, which can also be considered a “staying area”. The horizontal floor is made up of a deposit of very moist and pliable clay. This chamber is formed by large clastic blocks covered by calcite flowstones. The average relative humidity in this part of the cave is 99.9%, and the average temperature is 14.2°C (measured with OBO © pro v2). Between the two areas, there is a rough passage approximately 40 meters long, with very narrow crawlways (2 meters wide by 0.35 meters long), as well as places crossed by traversing (exerting pressure on the two walls) and small climbs of 2.5 meters. Some accidental torch impacts were tested in these areas. It must be stressed that the tests were performed outside the hibernation period of the bats that inhabit the cave (*Rhinolophus Euryale—*mediterranean horseshoe bat *-*, *R*. *ferrumequinum—*greater horseshoe bat-, *R*. *hipposideros—*lesser horseshoe bat -, and *M*. *schreibersii—*Bent-winged bat) (*S2 Appendix, detailed data about the experimental cave*).

## 3. Methodology: Testing lighting systems in Paleolithic caves

**Fig 5:**
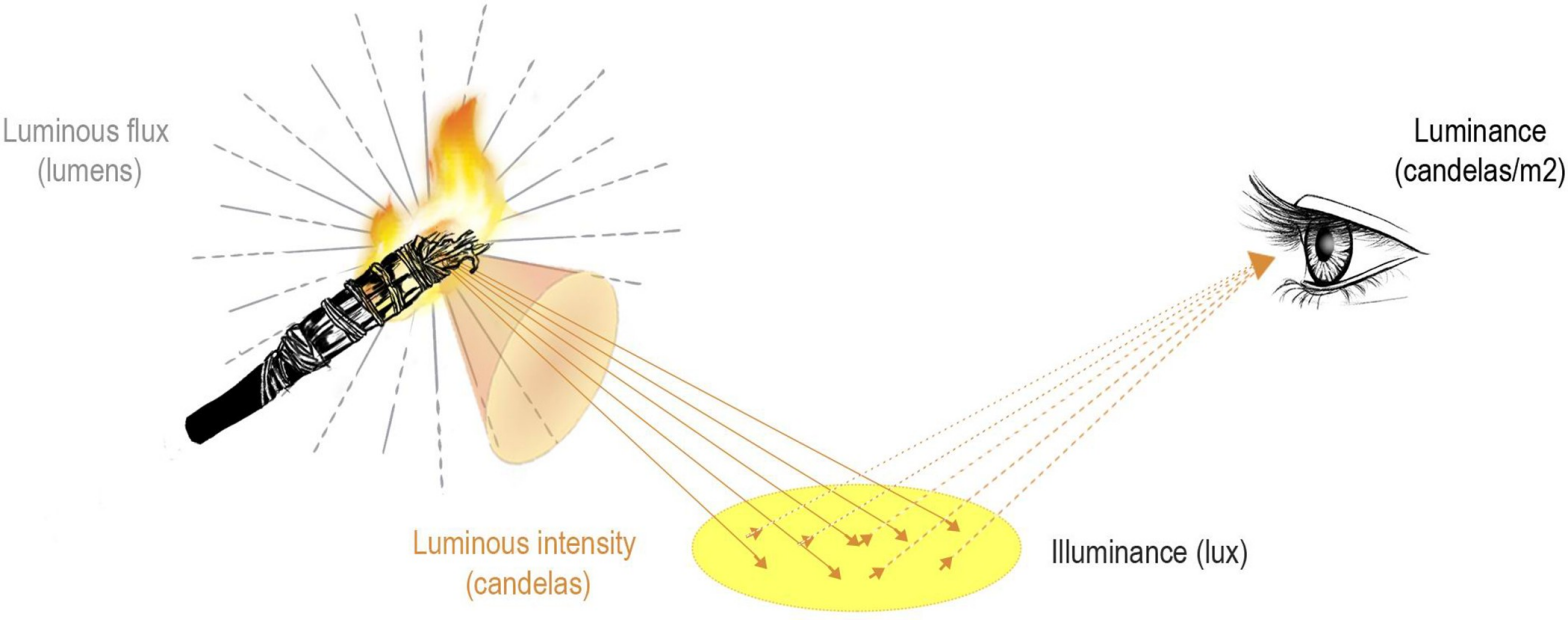
Scheme of the measured parameters.


Different physical parameters associated with the light radiation emitted by each experimental light resource were evaluated during the experimental activities:

- Duration: the length of time that the combustion emits luminous flux, that is, the time that the combustion has a flame. To measure this parameter, we used a basic stopwatch that recorded the hours, minutes, and seconds.
- Illuminance: the total amount of light received on a specific surface or point from a luminous flux (lumens) relative to the human eye. Its symbol is E, and it is measured in lux (lumen per m^2^) (Fig 5) [61]. This parameter was measured with a model PCE-L335 light meter (*S2 Appendix, detailed data on the instruments*). Measurements were made at different distances (every 10 cm) from the light source and with a 90° angle between the fire and the instrument.

$$1\;lux=1\;lm/m^{2}$$

- Luminous intensity: the amount of illuminance emitted in a certain direction. It is designated by the letter I, and its unit of measurement is the candle (cd) (Fig 5) [61]. We calculated this using the illuminance (lux) and the radius of action of the light.

I=ER2

- Action radius: minimum distance between the light source and total darkness (0 lux). To determine this parameter, we assume that the light intensity is the same in all directions, and a lux meter and a tape measure were used. The tape measure was placed on the floor, radially from the center of the light, and the illuminance was measured every 10 cm from the flame (following the tape measure until the light intensity was registered at 0 lux).

R=(L.)/E

- Luminance: This is a parameter that connects light intensity with the surface of the source. The concept of “source” is understood in a broad sense, which includes both primary sources (flame) and secondary sources (illuminated on the surface). The latter returns a certain degree of light, depending on its reflection coefficient. The unit of measurement for luminance is the candle per square meter (cd/m^2^) (Fig 5). We have applied a standard reflection coefficient for limestone (40%) [61], and its formula, based on illuminance, is as follows:

L=(rE)/π

We also provide information on the temperature reached by our lighting resource during operation. This parameter was measured using a model PCE-778 infrared thermometer (*S3 Appendix, detailed data about the measurement instruments*). This measurement should not be confused with the temperature attained by a fire’s flames. Furthermore, this could be used to determine the emitted light’s color temperature since the color temperature can be calculated based on flame temperatures and corresponds to the following formula Tk: Tk (degrees Kelvin) = Tc (degrees centigrade) + 273.

These parameters allowed us to characterize each light source quantitatively and, based on this, determine its potential and compare them. Besides, the appropriate type of lighting system for each type of underground space was proposed and linked with the different activities carried out at these sites, either the creation or visualization of Paleolithic art or cave exploration, among other possibilities. In this sense, we employed GIS software (ArcScene™ by ArcGis^®^) to recreate and evaluate the potential of quantitative results obtained from the experimental analysis. For this, we have worked directly with the 3D model of Sector J of Atxurra Cave [67] through a technique using Lines-of-Sight (LOS) analyses for complex three-dimensional sites [72]. This has allowed us to reach the first conclusions about Paleolithic illumination in a cave with rock art and illustrate the different ranges of the different lighting systems examined.

## 4. Results: Quantitative and qualitative data about Paleolithic lighting

Table 1 summarizes the measured parameters’ average values during the "useful life" of each source of light. Each experiment’s specific values during functioning can be found in the *S4 Appendix.* So, the materials used in the experimental program (and information on the conditions in which they were used), the manufacturing process of torches, lamps and fire, and the results of the control measures are presented in detail in the supplementary material. Providing this information makes the experiment replicable, which according to J. Baena-Preysler [73] is a basic Experimental Archaeology premise.

### 4.1. Torches

The onset of combustion was rapid in the torch experiments. Birch bark was useful for this purpose and was quickly consumed. However, it left few traces in the underground context since it volatilized after combustion, leaving scarce ashes.

The experimental torches were comfortable to transport and handle in the cave, and they radiated light in all directions. However, they produced smoke. Burning juniper and oak generated white smoke, whereas pine resin and birch bark produced denser black smoke; the latter burned quickly and lasted for a short time. Additionally, black smoke caused stains on the cave surfaces (soot) when the flame came too close to the cave walls (<5 cm).

**Fig 6:**
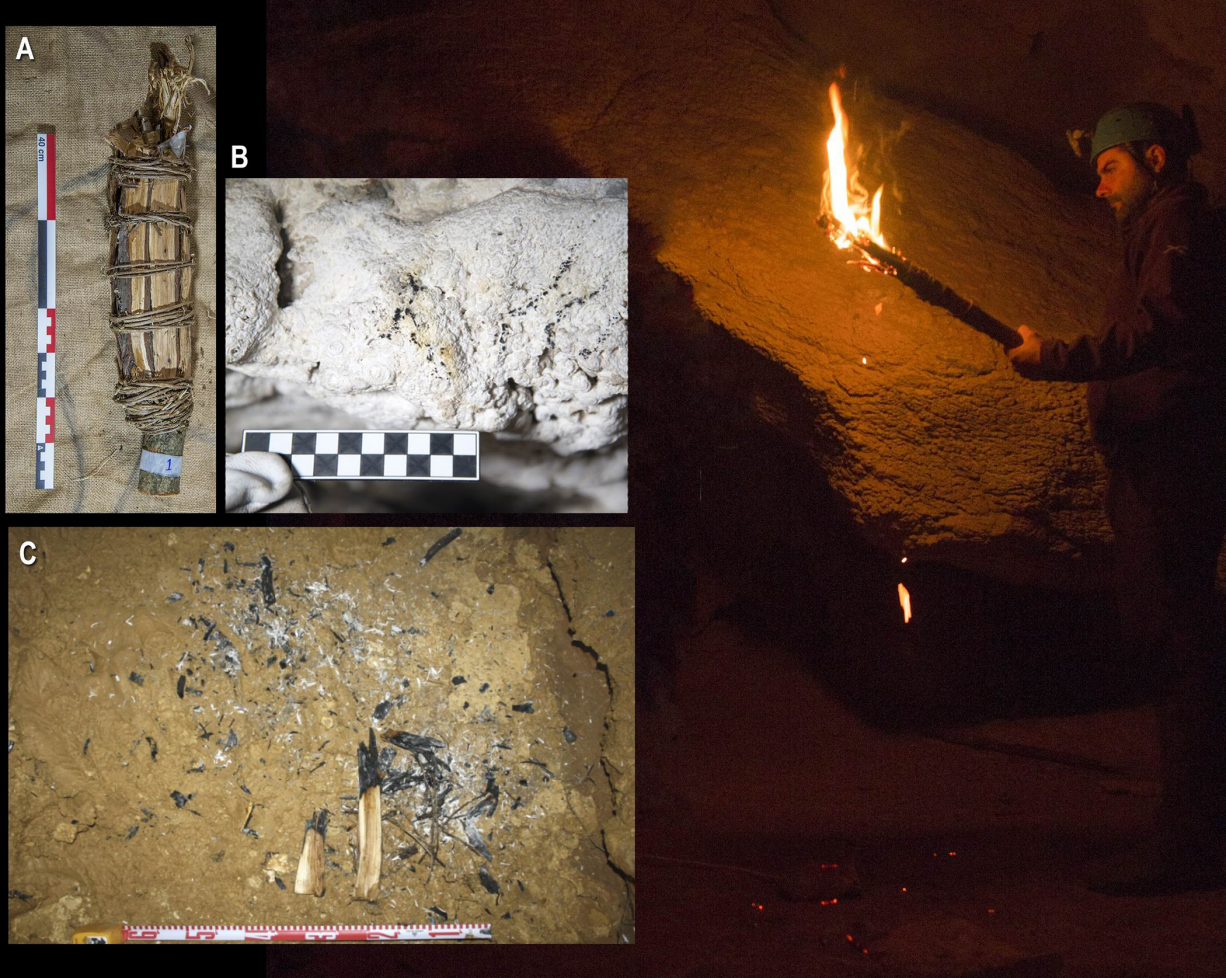
Photograph of Torch 1 in the experiment. Note how charred wood fell from it during its use. A. Torch 1 before being lit. B. Combustion marks left by the torch on the cave walls. C. Remains fell from the torch on the cave floor when it was being used.

The torches produced variable light intensity and functioned irregularly. This meant that the holder of the torch had to oxygenate it periodically and supervise it continuously. Fast transit favored combustion and oxygenated the torch. Oxygenation was also easily carried out by repeated semicircular movements with a certain intensity from left to right. After this action, the illuminance increased from 5 to 28 lux (measured at 20 cm) and 1.5 to 4.3 lux (measured at 40 cm) (*S4 Appendix, detailed data about experiment 3*). In this way, we managed to rekindle the torch on several occasions after its first extinction. We also observed that it was necessary not to over-assemble the components to obtain the torch’s rapid oxygenation. The addition of resin was very effective in maintaining an intense flame, although we observed that active combustion (more lighting) was detrimental to the prolonged use of the torch (*S4 Appendix, see experiment 2*). In contrast, animal fat extended the light’s duration since its combustion was slow and did not burn directly due to its impregnation in the wood (Fig 6A).

The intensity of light and the radius of action were directly correlated, i.e., high light intensity produced a larger radius of action. In contrast, the combustion temperature showed a more regular upward trend than the luminous parameters, and the temperature reached in the handle, in general, remained low. It allowed for comfortable transportation in a subterranean context.

The use of wooden torches in the underground landscape has principally produced two types of traces: charcoal and black marks. The charcoal pieces from the torches were heterogeneous in size; generally, less than 3 cm long, although larger portions separated occasionally. Their degree of carbonization was varied. Fully carbonized wood fragments separated from the torch in the pyrolysis stage; others with noncarbonized areas linked to the torrefaction stage; and others in a more advanced combustion process with the presence of ashes (Fig 6C). The distribution of these remains was scattered and occasional on the experimental cave’s floor, while some remains reached higher areas, such as shelves or interstices in the walls. Indeed, the oxygenation of the torch through sudden movements from side to side was the moment when the main quantity of charcoal was produced, as well as during the first moments of combustion. Certain charcoal accumulations were observed in areas (1–2 m^2^) where human transit stopped for some minutes (for example, in staying areas). Finally, in narrow spaces, direct and unintentional contact of the torches with the walls or ceilings of the cave occasionally occurred. It produced some black marks several centimeters in size on those spaces’ ceilings (Fig 6B).

Torch 5 (*S4 Appendix, experiment 5*) provided the most advantageous light characteristics for anthropic use of the cave, that is, the most minutes of light and greatest illuminance. In particular, the torch produced light (a flame) for 61 minutes, the average illuminance was 21.94 lux (measured 40 cm from the flame), the light intensity was 14.04 cd, the average radius of action light reached 2.47 meters, the average luminance was 2.79 cd/m, and the maximum temperature in the combustion was 633°C.

Torch 1 (*S4 Appendix, experiment 1*) produced luminous values similar to those of Torch 5, although the light production time was significantly lower, only 31 minutes. During the use of this torch, we observed that the flame tended to extinguish owing to poor oxygenation of the combustion nucleus, and therefore, the thick assembly structure could be conditioning its operation. As a result of this experiment, the next torches were built with a greater distance between their components. In turn, Torch 2 (*S4 Appendix, experiment 2*) burned very quickly, and the flame died out after 21 minutes. In this experiment, the amount of resin was disproportionate to the volume of woody fuel, which caused excessively rapid combustion. During its operation, the flame was constantly alive, and it was not necessary to relight it. Through both experiments, we determined the importance of appropriate oxygenation of the active part of the torch to obtain optimal functioning, neither too rapid (powerful flame but short use time) nor too slow (small flame and punctual extinction).

Torches 3 and 4 (*S4 Appendix, experiments 3–4*) were similar in their weight, components, and size. The only difference between the two was the wood’s drying time: Torch 4 was made with wood that had been dried for another 88 days. The latter’s illuminance, light intensity, and luminance values were higher, and radiation duration was longer. This indicates the suitability of dry wood for lighting. However, it could also affect the unpredictable behavior of the torches, which are notably susceptible to the movements of the person carrying them, their construction, and the physical characteristics of the underground environment (e.g., air currents and humidity).

### 4.2. Lamps

**Fig 7:**
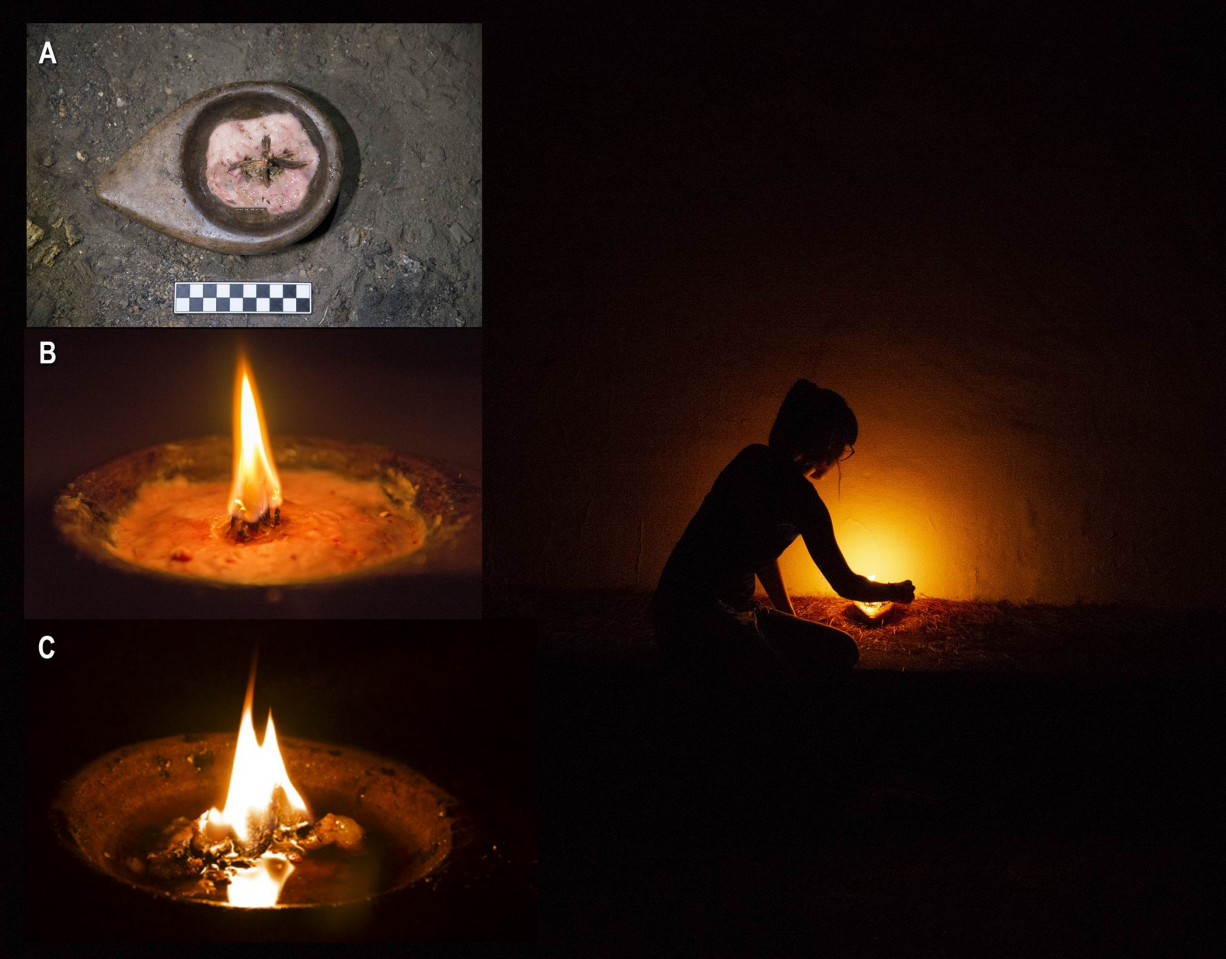
Photograph of experiment 6 with stone lamps. Observe the semicircular arc of light. A. Experiment 6 before being lit. B. Experiment 6 after functioning for 1’. C. Experiment 6 after functioning for 43’.

The animal fat lamps produced stable lighting for over an hour. Their luminous intensity was limited compared with torches and fireplaces, although enough for some functions (e.g., prolonged occupancy of the same place in a cave). Their radiation is not multidirectional. They emit a kind of semicircular halo; hence the light projected downwards is significantly less than that projected upwards and toward both sides. This is related to the small flame height relative to the lamp base’s size: the narrower the base and the higher the flame, the more light will be projected onto the floor. This lighting system does not produce smoke that could cloud and contaminate enclosed spaces. However, when pine resin was burning (and only when this fuel burned), the smoke added more pollution and blackening (Fig 7A–7C).

The flame is produced and maintained thanks to the absorption by capillarity of the fat in the lamp through the wick. Unlike resin, fat alone does not produce a flame but only through the wick. Therefore, in lamps with fatty fuel, the flame (and therefore the light) was strongly conditioned by the number of wicks, their height, their absorption capacity, and the degree of liquefaction of the fat. For this reason, the lamps reached their maximum efficiency after several minutes of operation. For example, this took place in experiment 6 after being alight for 20 minutes (*S4 Appendix, experiment 6*), when the fat was mostly melted. Likewise, it was necessary to maintain constant control over the wick to prevent it from sinking into the fatty fuel, causing the flame to be extinguished.

No combustion residue was produced by the lamps in the underground context and would only occur if there was a voluntary or involuntary fuel spill. Likewise, the combustion remains were scarce in the active part of the lamp and limited to a transparent film of fat, the partial carbonization of the branches that functioned as the wick, and some traces of smoke on the edge of the piece (caused by burning resin). There were no traces of rubefaction on the lamps in our experiments, probably because the highest temperatures barely exceeded 250°C (when this effect is produced by higher temperatures: ref. [74]).

Experiment 7 (*S4 Appendix, experiment 7*) presented a slightly higher illuminance, light intensity, luminance, and radius of action than experiment 6 (*S4 Appendix, experiment 6*). The average illuminance was 3.71 lux (measurements at 40 cm from the flame). The light intensity was 0.59 cd, the average radius of action the light reached was 1.57 meters, the average luminance was 0.47 cd/m, and the maximum temperature in the combustion was 176.33°C. This could be related to the addition of resin, which momentarily produces greater radiation. The temperature was also slightly higher in experiment 7, perhaps due to this type of fuel. In the lamp with mixed fuel (animal fat and pine resin), the illuminance reactivation was noticeable in the 40th minute. This occurred when a resin ball included in the fatty fuel was reached. The flame increased momentarily, but the lighting returned to its previous level after the resin was consumed. This is because of the terpenic hydrocarbons in the resin. These elements add energy to the combustion, producing quick ignition and power in the functioning, albeit for a short time.

### 4.3. Fireplace

**Fig 8:**
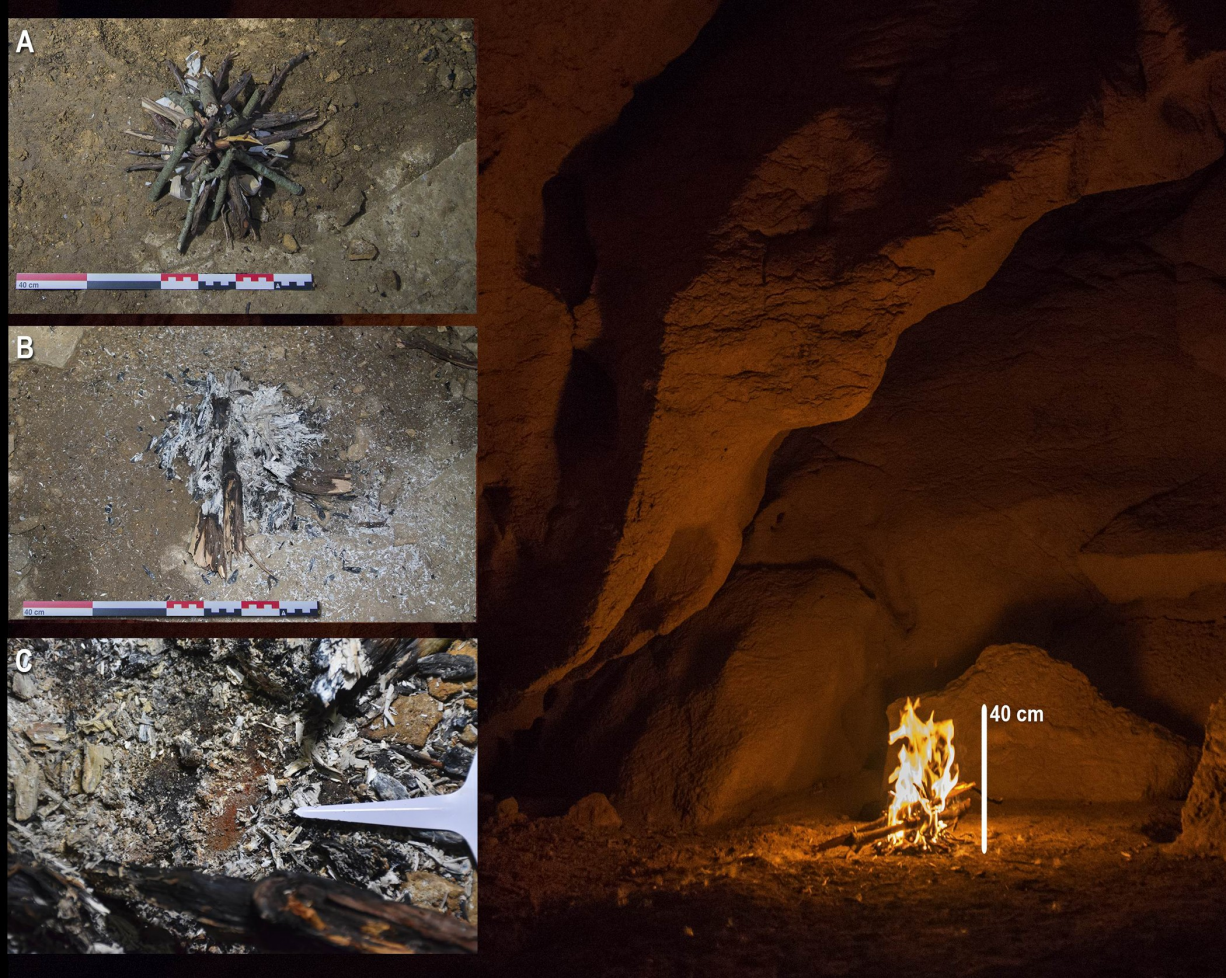
Photograph during experiment 8. A. Experiment 8 before combustion; vertical view. B. Experiment 8 after putting out the fireplace. C. Experiment 8 after the extinction of the fireplace, showing a detail of the rubefaction under the ashes.

The illumination, light intensity, and radius of action values increased compared to those in the previous experiment. The radius of action is highly variable, although it reaches high values (sometimes up to 4.5 meters); from two meters, illuminance values are close to zero (*S4 Appendix, experiment 8*). In particular, the average illuminance was 19.2 lux (measurements at 40 cm from the flame) (Fig 8), the light intensity was 3.07 cd, the average radius of action the light reached was 3.30 meters, the average luminance was 2.45 cd/m, and the maximum temperature in the combustion nucleus was 586.67°C. We also observed that the fireplace reached a maximum height of approximately 40–45 cm, and this did not cause smoke or rubefaction marks on the adjacent walls or ceiling (in any case, they were not close to the fireplace).

The fireplace produced white smoke, which quickly polluted the site. Therefore it was impossible to conclude the measurements until the fire was extinguished, and measurements were only taken for 30 minutes. We observed that the fireplace location was not appropriately placed regarding air currents; air currents in a cave are essential to achieving a prolonged stay underground [1]. However, in the case of large fires, convection currents are produced, and they would be efficient enough to evacuate gases outside of the cave [3].

Lastly, the combustion residues produced were ash, and charred wood remains (in different degrees of carbonization) superimposed on rubified sediment, all concentrated in a limited area (50 cm in diameter) (Fig 8A–8C).

## 5. Discussion

The Paleolithic lighting systems used for human frequentation in the inner areas of caves have diverse features. This should have conditioned their selection and use, depending on the duration and the activity to be carried out in the underground environment, as well as concerning the underground volume to be transited and the type of ventilation of the endokarst.

Wooden torches are the best lighting system for transiting wide spaces and exploring caves because they project light in all directions (illuminating the floor and the highest spaces correctly) [16, 51]. They are also easy to transport and do not dazzle the holder of the torch. They are also the best method for rapid movement due to their dynamism. According to our experimentation, their average action area is 5.98 meters in diameter, their average light intensity is 5 times greater than that of a grease lamp with two wicks, and their duration reaches an hour of operation. The area of action of the light (obtained through our experimentation) matches the values previously noted by P. Galant *et al*. [16]. Those authors indicated that torches produced an illuminated space between 4 and 6 m in length. However, some of our torches’ duration was longer than those obtained in previous experiments, as they gave an hour of light in some cases.

We have also confirmed that wood torches have a variable luminous flux and irregular functioning, which requires constant supervision and oxygenation actions. One of the main advantages of wood torches is the possibility of relighting them by oxygenation, without the need to bring them close to a fire after the first occasional extinctions. We managed to relight our torches by moving them from side to side repeatedly. Even human circulation with torches encourages active combustion through displacement oxygenation. In this sense, it was essential that the torch was made by several branches and not with a single stick, which would make oxygenation difficult. This feature is very useful for exploring caves since it means that the torch can be abandoned for a moment (for example, at an obstacle) without fear of its turning off, as it is possible to rekindle by sudden movement oxygenation.

As previously noted by other researchers [16] and as verified in our experiments, the impact of a torch against the wall has not been useful for relighting its flame (after extinction). The best method for this was to shake the tool in the air from side to side. Likewise, the torch releases charcoal as it is consumed. In our experiments, hitting the wall to eliminate the waste was unnecessary (again, several movements in the air were enough). In this sense, black parietal marks would be related to intentional spatial marks or involuntary impacts [12, 75] rather than actions connected with the torches’ functionality.

One of the main disadvantages of wooden torches is the production of smoke. This limits the stay in a site that is excessively enclosed or without good natural ventilation. Perhaps smaller torches would be better suited to smaller underground spaces since they would produce less smoke and use smoke-free alternative fuels (such as animal fat). However, if they are held high above the head, the smoke concentration in the space to be transited would be reduced, as smoke gathers mostly above the flame [2]. Their use time is also shorter than that of grease lamps. However, this could have been solved by carrying spare wooden torches ready to be ignited one after the other.

The combustion remains recorded during their operation are similar to those found in archaeological caves: scattered charcoal on horizontal surfaces in the caves and black marks on walls and ceilings. Interestingly, in our experiments, we recorded torch residues similar to those observed in the Réseau Clastres [13], consisting of a longitudinal and flattened semi-carbonized wood fragment detached from the torch before its complete carbonization (compare Figs 1B and 6C).

Grease lamps are the preferred lighting resource for use in small spaces over a long period. Their average light intensity in our experiments, measured at 1 meter, was similar to that of a candle, and their average diameter of action was 3.08 m. However, higher values are occasionally reached (*S4 Appendix, experiment 6*), and if more or larger wicks are added, the lighting could be more intense (and the fuel consumption faster). This system does not allow comfortable transit through the cave since it dazzles the person carrying the lamp and does not illuminate the floor well since it produces a semicircular light halo (Fig 6). Its duration is one of its main advantages since it greatly exceeds the hour of operation; another is that it does not produce smoke that could contaminate the space. It also functions regularly and does not require constant monitoring (unlike torches). It is only necessary to ensure that the wick does not sink into the liquid fuel once the fat melts. Supporting the wick on the lamp edge would solve this problem.

Our experimentation observed that combustion residues on the lamp and in the underground context are practically null. Therefore, the occasional use of a stone as a lamp will leave combustion residue that is practically negligible, which is a great difficulty for the characterization of Paleolithic lamps, as previously pointed out by other authors [11]. Only prolonged use, as well as a particular lithology of the stone, would cause the appearance of combustion traces such as rubefaction [70]. In this sense, a multi-analytical methodology for the proper characterization of these objects, through the determination of the combustion residue, especially charcoal and soot marks, has been recently proposed [19].

The lighting data obtained in experiment 6 can be compared with the data published by S.A. de Beaune [11], referring to a similar experiment (number 4: ref. 11, 126–127). The illuminance in that experiment, measured at a distance of 1 m from the flame, was 0.15 lux. Our lamp had an average illuminance value of 0.7 lux measured at a 1 m distance from the flame and between 5 and 50 minutes of functioning (*S4 Appendix*, *detailed data about experiment 6*). This difference could respond to the fact that the wicks used in the experiments are different. Her wick was made of a ball of lichen and moss, which stands out less from the liquid fuel than our wick made of a set of twigs arranged in a triangular pattern. As this researcher indicates, the state of the grease and the organization of the wick can greatly influence the efficacy of the lamp [10]. However, both experiments produce an average illuminance slightly higher than that of a full moon at night (0.12–0.25 lux) [61].

A fireplace is a static lighting system that makes it possible to stay in a specific area without carrying a light source. This allows specific activities to be carried out in the illuminated area. The combustion residues left by our experimental fireplace were similar to those found in some decorated caves, such as those found in Sector J (or the Ledge of the Horses) in Atxurra Cave [67], where there were three main concentrations of charcoals and ash on a thin layer of rubified clay. Our small experimental fireplace produced illumination similar to that of the experimental torches since we used a similar amount of woody fuel in both types of experiments (912 g). This illuminated an area with an average diameter of 6.60 meters for over half an hour. However, the size obtained from the mean of the radius of action and the illuminance is somewhat higher in the case of the fire than of the torches. Moreover, the production and concentration of smoke were higher than that for the torch experiments. This would be related to the fireplace’s static character, which determines that the concentration of gases falls on a smaller and more constant spatial volume. Here, the potential air currents present in a cave would play their role. In our experimental cave, there were no appreciable air currents. However, this could be different in the case of Sector J in Atxurra, since its situation in the middle of a long gallery, in which there are appreciable changes in air currents depending on the season of the year, could favor the evacuation of these gases.

In any case, illuminating capacity is correlated with the amount (and type) of fuel used, and the required use will condition the latter. For example, lighting a specific panel while decorating (or displaying) it, as in Atxurra [67], or other activities in deep zones [63], does not require large amounts of fuel. Our experimental fireplace showed that 500 g of juniper branches 1–2 cm in diameter and 400 g of dry oak branches 2–2.5 cm in diameter were sufficient for at least 30 minutes of activity (*S4 Appendix, experiment 8*). As in the case of torches, the main disadvantage of the fireplace is the production of smoke. If the space is enclosed or there is poor ventilation, there may be suffocating smoke, preventing any human stay. For this reason, this system will only be useful in well-ventilated areas [1]. To solve this problem, and as a hypothesis based on the numerical data obtained after the simulation of the fires in the Chauvet Cave, it has been observed that large fires with high flames favor the expulsion of gases through convection currents, as well as develop an optimal thermal stratification to allow human occupancy in a cave during the operation of the fire. Because polluting gases are deposited in the upper area of the chamber above the flame, they leave a toxicity-free space that is large enough to be walked by humans and is slightly crouched [2, 3, 49]. Following this interpretation, in Nerja Cave, we have suggested using supports (for example, speleothems) to lift the flame and concentrate the smoke in the upper part of the cave, using a smaller volume of wood fuel than in the previous case [19]. This issue could also be solved by combining wood fuel with other substances that produce less smoke, such as animal fat. Likewise, the balance between the endokarstic conduit’s size and the fire’s size must also be assessed (i.e., in a large chamber, the smoke emitted by a small fireplace would not affect the contamination of the cave too much).

The choice of an appropriate fuel for fire depends on its purpose [38, 69]. The physical characteristics of a fire constructed primarily for lighting are different from those used for smoking or cooking food. In the same way, the size of the cave or space to be lit, the type of cave passage, the action to be carried out (such as exploration or the creation of art), the number of people carrying lights, and the type of lighting system also must have influenced the choice of wood for the fuel. Furthermore, a single portable light might be sufficient to explore a large cave if it is accompanied by a clear exploration strategy (such as following the walls, memorizing particular speleothems or boulders, leaving waymarks, or marking the walls to indicate locations).

According to Théry-Parisot [69], the flame’s persistence depends on the degree of humidity in the wood: the lower the humidity is, the longer the duration. Additionally, using a wood type with essences, such as wood with terpenic hydrocarbons (resin), provides a taller flame. The environmental temperature and humidity also affect combustion. A high humidity level in a cave is not favorable (but not decisive) for reaching a high combustion temperature and consequently an intense fire. However, this might enable a flame of longer duration. High humidity is also a handicap when lighting fires. Therefore, a powerful tinder to start combustion, such as birch bark or resin (as used in our experiments), is important for lighting fires in the endokarst. The combustion structure’s morphology equally influences the flame’s intensity, as the speed and temperature of combustion increase with greater oxygenation. In this regard, the teepee-shaped structure worked optimally. In particular, in our experiments with torches and fireplaces, small caliber dry juniper and oak branches were appropriate as woody fuel for lighting activities.

For non-woody fuel, bone marrow and resin proved to be very appropriate substances for lighting that were complementary to each other. The former considerably increases the durability of the combustion of wood without producing smoke. The latter adds a rapid momentary increase in light, with the only handicap that it produces dense black smoke at that moment. The addition of resin would work similarly to the injection of water on the carbide stones used in caving in the last century, generating a momentary increase in the flame. The available archaeological data confirm the use of both substances for activities connected with lighting, at least in portable lamps, which combine resin (or resinous twigs) as the wick and animal fat as the main fuel [11].

Similarly, our experimentation has demonstrated that wooden torches and marrow lamps are complementary for lighting caves. The former are suitable for moving through the cave (multidirectional radiation of light and easy to carry), while the latter are suitable for a prolonged stay in an enclosed space with little ventilation (longer duration of light and absence of smoke). A system combining the two lighting methods would be an optimal resource in a cave. This system could have a long shape as a torch so that it could be easy to carry and the light could be projected in all directions, while it should operate in a similar way to a lamp by the absorption of bone marrow fuel. This could contribute to greater durability and the absence of smoke. *S5 Appendix* presents an example of a torch lamp that we manufactured. However, numerous alternatives might exist, creating new experimental possibilities to be tested in the future. In any case, as with lamps, this system would not leave combustion residues, and the fact that it is made of perishable materials (unlike portable lamps) makes it impossible to check whether alternatives similar to this were used in the Upper Paleolithic or not.

Bearing in mind the mean values of the intensity of light obtained in our experimentation, Paleolithic lighting systems would provide mesopic vision. This confirms that human visual perception underground was related to color less than to the contrast between illuminated and unilluminated areas and the play of light and shadows. However, some colors could be perceived, as the cones were active, albeit partially. Long-wavelength colors (red, orange, and yellow) would be best perceived in these conditions. Indeed, warm colors would be emphasized, owing to the low color temperature emitted by the light from fires (≈1000–3000 K).

**Fig 9:**
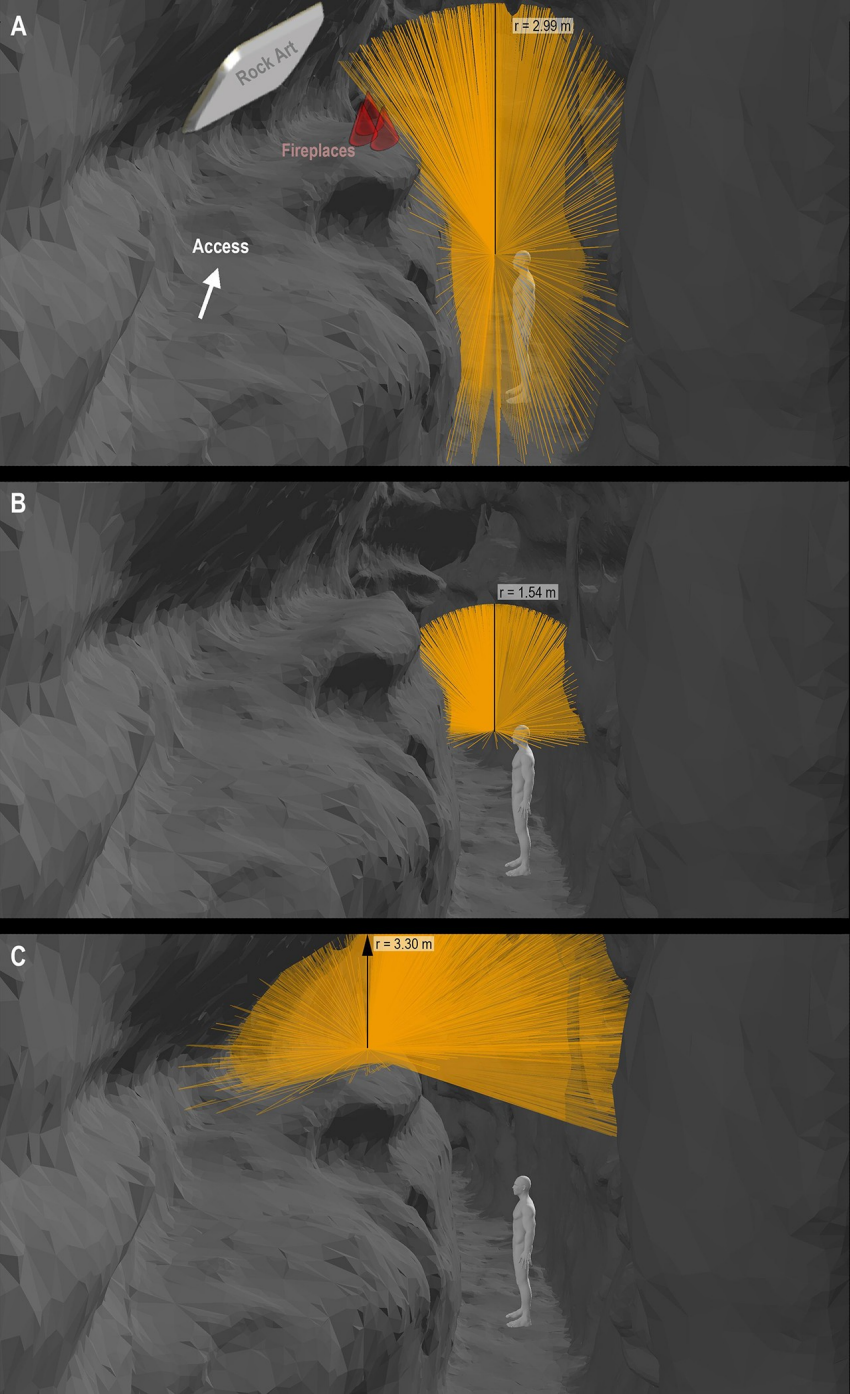
Representation of the range of the illuminance (lux) of the three analyzed lighting systems. A. Wood torch. B. Portable grease lamp. C. Fireplaces with wood fuel. (Measurements made in ArcScene™ by ArcGis^®^ based on the data from the experiments).

These were applied through software for spatial analysis (ArcScene™ by ArcGis®) in a Paleolithic cave regarding the radius of action. For this, a virtual 3D model of the Atxurra cave was used, specifically of Sector J, also called the Ledge of the Horses [64]. In this sector, a natural platform elevated 2.5 m above the passage floor was found, with 84 graphic units placed in two panels. They include approximately 50 animal engravings (some of them also painted in black) in Upper Magdalenian style, with bison, goats, horses, and hinds, many of them overlapping. In this ledge, hundreds of scattered charcoals, lithic tools, and five areas of reddened clay with ashes and charcoals were also identified, belonging to three probable fireplaces (dated by C14-AMS in the Upper Magdalenian) [67]. For the spatial analysis, we displayed the three analyzed lighting systems. A torch and a lamp were placed in the lower part of the gallery at the height of 1.66 m, the estimated average stature during the final Upper Paleolithic [76, 77]. Moreover, the fireplaces were also geolocated in the replica. From them, we carried out a Lines-of-Sight (LOS) analysis in a closed environment [72] to analyze the space. The radius of action for the area each illumination system would occupy was then estimated, after taking the cave’s natural morphologies into account (Fig 9).

The results show some interesting implications since it is observed that the decorated panels would be barely perceptible from the lower parts of the gallery, regardless of whether torches or lamps were carried. Reflectance could increase the range of these tools, but the rock art would not be clearly observable if it were not illuminated from the top of the ledge. However, the fireplaces seem to be strategically arranged as they illuminate the entire decorated space. The entrances to reach this decorated sector are also illuminable using a torch. It does not seem by chance that the optimal routes estimated to access this space are covered with scattered charcoals (surely fallen from the torches used in the Magdalenian period).

Finally, our experiments’ numerical estimations have been very useful to evaluate other insights related to accessibility. For example, in an experiment performed to measure the effect of the morphologies of the cave in the transit through it [70], it was observed that the lighting greatly conditioned the maximum achievable speed: in the widest galleries, where it would be possible to move faster, it was limited to an average of 1.23 meters per second because the limited radius of action of a torch (an estimated value of 2.99 meters) made it impossible to illuminate at a sufficient distance to acquire higher speed safely.

It was also estimated that a minimum of 38.39 minutes is required to reach Sector J of Atxurra [70]. Suppose we interrelate this information with our results on a torch’s duration (preferential light resource in this cave identified through internal archaeological context). In that case, we can determine that, as a minimum, it would have been necessary to carry a spare torch to access this part of the cavity, ensuring the return. This would imply a minimal cost of effort involving the harvesting and transport of at least 1.82 kg of dry juniper wood, along with a total of 140–170 g of complementary fuels (birch bark, ivy, bovid or deer marrow, or pin resin). These data are a minimum value since they only take into account the direct transit toward Sector J. If we add the execution (or visualization) of the rock art or the visit or exploration of other areas in the same cave (among other possible ritual activities), this value would increase.

From future perspectives, we consider it essential to continue investigating this matter both in archaeological sites and experimental studies. Only with a large corpus of archaeological remains, including different types of lighting systems (and fuels), studied through an interdisciplinary approach will it be possible to adequately reproduce Paleolithic light resources, which allows holistic archaeological interpretations of underground activities to be achieved, as well as reproduce and determine how those groups perceived the underground landscape.

## 6. Conclusion

In this work, the main lighting systems (torches, grease lamps, and fireplaces) used during the Paleolithic period to enter the depths of the caves were qualitatively and quantitatively characterized: their duration, illuminance, luminous intensity, the radius of action of the light, and the temperature reached by the woody fuel were measured accurately. Likewise, each lighting system’s characteristic combustion residues were identified to achieve a better identification for the archaeological record. In addition, the luminous data were analyzed through GIS technology and represented within the 3D model of the Atxurra cave, an underground space frequented during the Paleolithic. With this, we manage to illustrate each resource’s luminous peculiarities in these contexts and delve into the implications of the characterization of Paleolithic lighting in the archaeological studies of these caves.

The replicated lighting resources have been based on a comprehensive collection of available archaeological data on this topic from various caves in Southwestern Europe. Each lighting tool has different physical characteristics in terms of light intensity, the radius of action of light, smoke production, and direction of radiation. These parameters would have conditioned the choice of one system or another based on the features of the underground space to be traveled, its volume and level of ventilation, as well as the duration and type of activity to be carried out.

In particular, we highlight the complementary of lamps enlivened with animal grease and torches for lighting the underground environment. A resource midway between the two lighting systems could be an optimal light source for Paleolithic underground activity (combining the lamps’ durability and smokeless nature and the radiation system and the convenience of carrying the torches). We also emphasize the complementary nature of resin and animal fat for lighting. The former provides greater punctual intensity when necessary, and the latter provides great durability through lower fuel consumption.

In addition, and for the specific case of torches, it has been shown that the construction of the assembly from several thin branches (following archaeological data), and not a single central mast, is the best way to build this tool to maintain a long-living flame and allow it to reignite through aerial movements after its first extinctions.

In any case, our experiments on Paleolithic lighting point to planning in the human use of caves in this period and the importance of lighting studies to unravel the activities carried out by our ancestors in the deep areas of caves.

## Supporting information

S1 Appendix Anthracological data from Paleolithic light systems (torches and fires). (PDF)

S2 Appendix Experimental cave data. (PDF)

S3 Appendix Measured tools data. (PDF)

S4 Appendix Experiment data. (PDF)

S5 Appendix Model torch-lamp. An idea.
(PDF)

## References

[1] Kedar Y, Barkai R. The significance of air circulation and hearth location at Paleolithic Cave Sites. Open Quat. 2019;5(1). doi: 10.5334/oq.52

[2] Lacanette D, Mindeguia JC, Brodard A, Ferrier C, Guibert P, Leblanc JC, et al. Simulation of an experimental fire in an underground limestone quarry for the study of Paleolithic fires. Int. J. Therm. Sci. 2017;120: 1–18.

[3] Salmon F, Lacanette D, Mindeguia JC, Sirieix C, Bellivier A, Lebranc JC, et al. Localized fire in a gallery: Model development and validation. Int. J. Therm. Sci. 2019;139: 144–159.

[4] Álvarez M., Fiore D. Recreando imágenes: diseño de experimentación acerca de las técnicas y los artefactos para realizar grabados rupestres. Cuadernos del Instituto Nacional de Antropología y Pensamiento Latino Americano, 1995;16: 215–240.

[5] Medina-Alcaide MA, Garate D, Ruiz-Redondo A, Sanchidrián JL. Beyond art: The internal archaeological context in Paleolithic decorated caves. J. Anthrop. Archaeol. 2018;49: 114–128.

[6] Jaubert J, Verheyden S, Genty D, Soulier M, Cheng H, Blamart D, et al. Early Neanderthal constructions deep in Bruniquel Cave in southwestern France. Nature 2016b;534(7605): 111–114. 27251286 10.1038/nature18291

[7] Aranburu A, Arsuaga JL, Sala N. The stratigraphy of the Sima de los Huesos (Atapuerca, Spain) and implications for the origin of the fossil hominin accumulation. Quat. Int. 2017;433: 5–21.

[8] Dirks PH, Berger LR, Roberts EM, Kramers JD, Hawks J, Randolph-Quinney PS, et al. Geological and taphonomic context for the new hominin species *Homo naledi* from the Dinaledi Chamber, South Africa. eLife 2015;4: e09561. doi: 10.7554/eLife.09561 26354289 PMC4559842

[9] Lari M, di Vincenzo F, Borsato A, Ghirotto S, Micheli M, Balsamo C, et al. The Neanderthal in karst: First dating, morphometric, and paleogenetic data on the fossil skeleton from Altamura (Italy). J. Hum. Ev. 2015;82: 88–94. 10.1016/j.jhevol.2015.02.007 25805042

[10] Onac BP, Viehmann I, Lundberg J, Lauritzen SE, Stringer C, Popita V. U-Th ages constraining the Neanderthal footprint at Vârtop Cave, Romania. Quat. Sci. Rev. 2005;24: 1151–1157.

[11] Beaune SA. Lampes et godets au Paléolithique. Supplément à Gallia Préhistoire 23. Paris: CNRS; 1987.

[12] Beaune SA. Les techniques d’éclairage paléolithiques: un bilan. Paléo, Revue d’Archéologie Préhistorique 2000;12: 19–27.

[13] Clottes J, Simonnet R. Retour au Réseau Clastres (Niaux, Ariège). Prehist. Ariégeoise 1990;45: 51–139.

[14] Clottes J. Les cavernes de Niaux. Art Préhistorique en Ariège. Paris: Seuil; 1995. doi: 10.1111/j.1432-1033.1995.160_c.x

[15] Ambert P, Galant P, Guendon J, Colomer A, Dainat D, Beaumes B, et al. Les gravures et les empreintes humaines de la grotte d’Aldène (Cesseras-Hérault) dans leur contexte chronologique et culturel. Bull. Mus. Anthrop. Préhist. Mon. 2007;47: 3–36.

[16] Galant P, Ambert P, Colomer A, Guendon JL. Les vestiges d’éclairages préhistoriques de la galerie des Pas de la grotte d’Aldène (Cesseras Hérault). BMAPM 2007;47: 37–80.

[17] Milner N, Taylor B, Conneller C. Star Carr Volume 1–2. York: White Rose University Press; 2018.

[18] Padilla JA, editor. El reino de la sal. 7.000 años de historia de Hallstatt. Catálogo de la exposición, Museo Arqueológico de Alicante, Naturhistorisches Museum Wien. Alicante: Diputación de Alicante; 2013.

[19] Medina-Alcaide MA, Cabalín LM, Laserna J, Sanchidrián JL, Torres AJ, Cosano S, et al. Multianalytical and multiproxy approach to the characterization of a Paleolithic lamp. An example from Nerja cave (South of Iberian Peninsula). J. Archaeol. Sci. Rep. 2019;28. doi: 10.1016/j.jasrep.2019.102021

[20] Bégouën R, Pastoors A, Clottes J. La Grotte d’Enlène: Immersion dans un habitat magdalénien. Paris: Association Louis Begouën/In Fine éditions d’art; 2019.

[21] Ferrier C, Debard E, Kervazo B, Brodard A, Guibert P, Baffier D., et al. Les parois chauffées de la grotte Chauvet-Pont d’Arc (Ardèche France): caractérisation et chronologie. Paléo 2014;25: 59–78.

[22] Ferrier C, Bellivier A, Lacanette D, Leblanch JC, Mindeguia JC, Salmon F. L’utilisation du feu dans l’endokarst au Paléolithique: approche interdisciplinaire et expérimentale (programme CarMoThaP). Karstologia 2017;70: 23–32.

[23] Guibert P, Brodard A, Quiles A, Geneste JM, Baffier D, Debard E et al. When were the walls of the Chauvet-Pont d’Arc Cave heated? A chronological approach by thermoluminescenca. Quat. Geochronol. 2015;29: 36–47.

[24] Delluc B, Delluc G. L´éclairage. In: Leroi-Gourhan A, Allain J, editors. Lascaux inconnu. Paris: CNRS; 1979. pp. 121–142.

[25] Baffier D, Girard M. Les cavernes d’Arcy-sur-Cure. Paris: La Maison des Roches; 1998.

[26] Cortés M, Simón MD, Morales A, Lozano MC, Vera JL, Odriozola CP. La caverna iluminada: una singular lámpara gravetiense arroja luz sobre el arte parietal de la cueva de La Pileta (Benaoján, Málaga). TP 2016;73(1): 115–127.

[27] Perlès C. Préhistoire du feu. Paris: Masson; 1997.

[28] Rigaud A. Étude expérimentale des lampes de La Garenne (Indre). Revue archéologique du Centre de la France 2000;39: 215–221.

[29] Allain J. Les lampes magdaléniennes de Saint-Marcel. In: Congrès préhistorique de France. 16ª sesión. Paris: CNRS; 1965. pp. 178–183.

[30] Garate D, Tapia J, Rivero O, Álvarez I, Abendaño V, Aranburu A. Alkerdi 2: a new Gravetian rock art cave in the Western Pyrenees. INORA 2017;79: 10–12.

[31] Ragland KW, Aerts DJ, Baker AJ. Properties of wood for combustion analysis. Bioresource technol. 1991;37(2): 161–168.

[32] Szubel M, Filipowicz M, Goryl W, Basista G. Characterization of the wood combustion process based on the TG analysis. Numerical Modelling and Measurements Performed on the Experimental Stand. E3S Web Conf. 2016; 10: 133.

[33] Théry-Parisot I, Thiébault S. Le pin (Pinus sylvestris): préférence d’un taxon ou contrainte de l’environnement? Étude des charbons de bois de la grotte Chauvet. BSPF 2005;102(1): 69–75.

[34] Medina-Alcaide MA, Sanchidrián JL, Zapata L. Lighting the dark: Wood charcoal analysis from Cueva de Nerja (Málaga, Spain) as a tool to explore the context of Palaeolithic rock art. CR Palevol. 2015;14(5): 411–422.

[35] Théry-Parisot I, Thiébault S, Delannoy JJ, Ferrier C, Feruglio V, Fritz C et al. Illuminating the cave, drawing in black: wood charcoal analysis at Chauvet-Pont d’Arc. Antiquity 2018;92(362): 320–333.

[36] Badal E. L’anthracologie préhistorique: à propos de certains problèmes méthodologiques. Bulletin de la société botanique de France. Actualités Botaniques 1992;139(2–4): 167–189.

[37] Asouti E, Austin P. Reconstructing woodland vegetation and its exploitation by past societies, based on the analysis and interpretation of archaeological wood charcoal macro-remains. Environ. Archaeol. 2005;10 (1): 1–18.

[38] Théry-Parisot I, Chabal L, Chrzavzez J. Anthracology and taphonomy from wood gathering to charcoal analysis. A review of the taphonomic processes modifying charcoal assemblages, in archaeological contexts. Palaeogeogr., Palaeoclimatol., Palaeoecol. 2010;291(1–2): 142–153.

[39] Moskal-del Hoyo M, Ntinou M, Carrión Y, Vidal-Matutano P, Badal E. Palaeolithic and Neolithic wood charcoal remains as important tools for chronological, ethnographic and environmental studies. In: Valde-Nowak P, Sobczyk K, Nowak M, Zralka J,editors. Multas per gentes et multa per saecula. Amici magistro et college suo Ioanni Christopho Kozlowski dedicant. Poland: Institute of Archaeology, Jagiellonian University; 2018. pp. 593–600.

[40] Henry A, Théry-Parisot I. From Evenk campfires to prehistoric hearths: charcoal analysis as a tool for identifying the use of rotten wood as fuel. Journal of Archaeological Science 2014;52: 321–336.

[41] Henry A, Zavadskaya E, Alix C, Kurovskaya E, Beyries S. Ethnoarchaeology of Fuel Use in Northern Forests: Towards a Better Characterization of Prehistoric Fire-Related Activities. Ethnoarchaeol. 2018;10(2): 99–120.

[42] Leroi-Gourhan A, Schweingruber FH, Girard M. Les bois. In: Leroi-Gourhan A, Allain J editors. Lascaux inconnu. Paris: CNRS; 1979. pp. 185–188.

[43] Ramos J, Weninger GC, Cantalejo P, Espejo MM. Cueva de Ardales (Málaga): intervenciones arqueológicas, 2011–2014. Málaga: Pinsapar; 2014.

[44] Berthelot M. Sur une lampe préhistorique trouvé dans la grotte de La Mouthe. Comptes Rendus. Acad. Sci. 1901; 666.

[45] Newcomer MH. Stone-carving with flint: experiments with a Magdalenian lamp. Staringia 1981;6(1): 77–79.

[46] Pérez E, Muñoz D. Los combustibles en las lámparas del Paleolítico superior. BAEX 2015;10: 197–208.

[47] Ruiz-González D, Piedrabuena S, Jiménez M. ¡Y se hizo la luz! El potencial de la médula ósea como combustible para la iluminación en sociedades prehistóricas. Poster, V Congreso de Arqueología Experimental (25–27 octubre 2017). Tarragona: IPHES, ICAC, ICRPC; 2017

[48] Pitchford L. Lighting The Art—An investigation into European Upper Palaeolithic Lamp Technology through Experimental Archaeology and 3D modelling. Oral communication, 12th Experimental Archaeology Conference EAC12, World Tour; https://www.youtube.com/watch?v=BXm9rIbwjsY&t=1s

[49] Salmon F, Ferrier C, Lacanette D, Mindeguia JC, Leblanc JC, Fritz C, Sirieix C. Numerical Reconstruction of Paleolithic Fires in the Chauvet-Pont d’Arc Cave (Ardèche, France). Journal of Archaeological Method and Theory 2020; 10.1007/s10816-020-09484-5.

[50] Malvesin-Fabre G, Parriaud H. Une hypothèse sur l’éclairage des grottes au Paléolithique. Actes du XIV Congrès Préh. de France (Strasbourg-Metz, 1953); 1955. pp. 426–430.

[51] Rouzaud F. Fréquentation humaine dans le monde souterrain durant la Prehistoire. Actes du II Colloque sur le patrimoine troglodytique; 1990. pp. 29–31.

[52] Roussot A. Review of "Les Cavernes de Niaux" by J. Clottes. Bull. Soc. Ariège-Pyrénées 1995; 50: 315–23

[53] Moyes H. Charcoal as a proxy for use-intensity in ancient Maya cave ritual. In: Fogelin L, editor. Religion, archaeology, and the material world. Carbondale: Centre for Archaeological Investigations, Southern Illinois University; 2008. pp.139–158.

[54] Hoare S. Assessing the Function of Palaeolithic Hearths: Experiments on Intensity of Luminosity and Radiative Heat Outputs from Different Fuel Sources. J. Paleolit. Archaeol. 2020. doi: 10.1007/s41982-019-00047-z

[55] Théry-Parisot I. Fuel management (bone and wood) during the Lower Aurignacian in the Pataud rock shelter (Lower Palaeolithic, Les Eyzies de Tayac, Dordogne, France). Contribution of experimentation. Journal of Archaeological Science 2002;29(12): 1415–1421.

[56] Théry-Parisot I, Costamagno S. Propriétés combustibles des ossements: Données expérimentales et réflexions archéologiques sur leur emploi dans les sites paléolithiques. Gallia Préhistoire 2005;47: 235–254.

[57] Yravedra J, Baena J, Arrizabalaga A, Iriarte MJ. El empleo de material óseo como combustible durante el Paleolítico Medio y Superior en el Cantábrico. Observaciones experimentales. Museo de Altamira, Monografías 2005;20. pp. 369–383.

[58] Costamagno S, Théry-Parisot I, Brugal JP, Guibert R. Taphonomic consequences of the use of bones as fuel. Experimental data and archaeological applications. In: O’Connor T, editor. Biosphere to Lithosphere, Proceedings of the 9th Conference of the International Council of Archaeozoology. Oxford: Oxbow books; 2015. pp. 51–62

[59] Beresford-Jones DG, Johnson K, Pullen AG, Pryor AJ, Svoboda J, Jones MK. Burning wood or burning bone? A reconsideration of flotation evidence from Upper Palaeolithic (Gravettian) sites in the Moravian Corridor. Journal of Archaeological Science 2010;37(11): 2799–2811.

[60] Vanlandeghem M, Desachy B, Buonasera T, Norman L, Théry-Parisot I, Carré A, et al. Ancient arctic pyro-technologies: Experimental fires to document the impact of animal origin fuels on wood combustion. Journal of Archaeological Science: Reports 2020;33: 10.1016/j.jasrep.2020.102414

[61] Comité Español de la Iluminación (C.E.I.). El libro blanco de la iluminación. Madrid: C.E.I.; 2011.

[62] Harten HU. Physik für Mediziner. Berlin: Springer; 1997.

[63] Pastoors A, Weniger GC. Cave Art in Context: Methods for the Analysis of the Spatial Organization of Cave Sites. J. Archaeol. Res. 2011;19(4): 377–400.

[64] Hoyos A. Color e illusion. CES odontología 2009;14(2): 53–62.

[65] Delluc B, Delluc G. Eye and vision in paleolithic art. In: Bahn P.G., editor. An Enquiring Mind. Studies in Honor of Alexander Marshack: Oxbow Books, Oxford/Oakville; 2009. pp. 77–98.

[66] Griefahn B. Arbeitsmedizin. Stuttgart: Thieme; 1996. doi: 10.1080/10803548.1996.11076338

[67] Garate D, Rivero O, Ríos-Garaizar J, Arriolabengoa M, Medina-Alcaide MA, Ruiz-López JF, Intxaurbe I, et al. The cave of Atxurra: a new major Magdalenian rock art sanctuary in Northern Spain. J. Archaeol. Sci. Rep. 2020;29: 102–120.

[68] Carrión JS, Finlayson C, Férnández S, Finlayson G, Allué E, López-Sáez JA, et al. A coastal reservoir of biodiversity for Upper Pleistocene human populations: Palaeoecological investigations in Gorham’s Cave (Gibraltar) in the context of the Iberian Peninsula. Quat. Sci. Rev. 2008;27: 2118–2135.

[69] Théry-Parisot I. Economie des combustibles au paléolithique: expérimentation, taphonomie, anthracologie. Paris: CNRS; 2001.

[70] Intxaurbe I, Arriolabengoa M, Medina-Alcaide M.A, Rivero O, Rios-Garaizar J, Salazar S, Libano I, Garate D. Quantifying accessibility to Palaeolithic rock art: methodological proposal for the study of human transit in Atxurra Cave (Northern Spain). Journal of Archaeological Science 2021;125: 10.1016/j.jas.2020.105271

[71] Rouzaud F. La Paleoespeleologie. L’homme et le milieu souterrain pyreneen au Paleolithique Superieur. Toulouse: CNRS et départements de la Haute-Garonne et de l’Ariège; 1978.

[72] Landeschi G, Dell’Unto N, Lundqvist K, Ferdani D, Campanaro D.M, et al. 3D-GIS as a platform for visual analysis: investigating a Pompeian house. Journal Archaeological Science 2016;65: 103–113.

[73] Baena-Preysler J. Arqueología Experimental. Algo más que un juego. Boletín de Arqueología Experimental 1997;1: 3–5.

[74] Canti MG, Linford N. The effects of fire on archaeological soils and sediments: temperature and colour relationships. PPS Cambridge University Press 2005;66: 385–395.

[75] Le Guillou Y. Circulations humaines et occupation de l’ espace souterrain à la grotte Chauvet-Pont- d’Arc Fréquenmtation humaines et art parietal. In: Geneste JM, editor. La grotte Chauvet à Vallon-Pont- d’Arc: un bilan des recherches pluridisciplinaires. Paris: BSPF; 2005. pp. 117–134.

[76] Holt BM. Mobility in Upper Palaeolithic and Mesolithic Europe: evidence from the lower limb. American Journal of Physical Anthropology: The Official Publication of the American Association of Physical Anthropologists 2003;122(3): 200–215. 10.1002/ajpa.10256 14533179

[77] Cox S.L, Ruff C.B, Maier R.M, Mathieson I. Genetic contributions to variation in human stature in prehistoric Europe. Proceedings of the National Academy of Sciences 2019;116(43): 21484–21492. doi: 10.1073/pnas.1910606116 31594846 PMC6815153

