# Supplementary material for: The conquest of the dark spaces: An experimental approach to lighting systems in Paleolithic caves
Source: PLoS One. 2021 Jun 16;16(6):e0250497. doi: 10.1371/journal.pone.0250497 (PMC8208548; doi:10.1371/journal.pone.0250497)
Supplement: S1 Appendix — (PDF) [file pone.0250497.s001.pdf]

**<u>*S1 Appendix*: Anthracological data from Paleolithic light systems (torches and fires).</u>**

In the next table, we present the anthracological data from Paleolithic caves and Paleolithic lighting systems (torches and fires). In particular, we detail the site's name, the geographical localization, the chronology of the Paleolithic illumination remains, the number of charcoals analyzed, the taxonomic information, and the references.

| CAVE | CHRONOLOGY | N° OF CHARCOALS | TAXA PRESENT | REFERENCE |
|---|---|---|---|---|
| **BÀSURA (Savona, Italy)** | Upper Magdalenian | 2 | *Pinus tp. sylvestris/mugo* (2) | 78 |
| **CHAUVET (Ardèche, France)** | Aurignacian and Gravettian | 293 | *Pinus nigra/sylvestris* (292), cf. *Rhamnus* (1) | 33; 79; 35 |
| **COMTE (Valencia, Spain)** | Gravettian | 238 | *Acer* sp. (1), *Juniperus* sp. (107), *Leguminosae* (1), *Pinus halepensis* (1), *Pinus nigra-sylvestris* (116), *Pinus* sp. (3), *Prunus* sp. (8) and *Quercus* sp. *Deciduous* (1) | 80; Personal communication Y.Carrión Marco |
| **COSQUER (Marseille, France)** | Aurignacian, Gravettian and Solutrean (Ancient and Middle) | 258 | *Pinus sylvestris* (142)<br>Conifer (116) | 81; 82 |
| **CUSSAC (Aquitaine, France)** | Gravettian | 1 | *Juniperus* sp. (1) | 79 |
| **ETXEBERRI (Pyrénées-Atlantiques, France)** | Middle Magdalenian | 1 | 1 Gymnosperm (*Pinus* sp. or *Juniperus* sp.) | 83; Personal communication L. Zapata Peña |
| **LA GARMA (Cantabria, Spain)** | Middle Magdalenian | 1 | *Juniperus* sp. | 85 |
| **LASCAUX (Dordogne, France)** | Lower Magdalenian level | 233 | *Abies pectinata* (1), *Quercus* sp. (deciduous leaf, thick branch) (83), *Juniperus* sp. (thin branches) (99), *Salix* sp. (5)., cf. Salix-Populus (8), *Pinus pinaster* (7), *Corylus* sp. (10), *Betula* sp. (6), *Alnus* glutinosa (4), cf. *Castanea* (4), cf. *Prunus* (1), *Monocotiledonea* herbácea (2), *Pinus* tp. sylvestris (1), *Taxus baccata* (1), *Gramineae* (1) | 42 |
| **MORGOTA (Basque Country, Spain)** | Upper Magdalenian | 22 | *Juniperus* sp. (7), Conifer (6), Deciduous Quercus (6), Angiosperm (3) | 86 |
| **MOULIN DE LAGUENAY (Corrèze, France)** | Gravettian | 1 | *Salix* sp. (1) | 87 |
| **NERJA (Andalusia, Spain)** | Upper Paleolithic | 8 | *Pinus* tp. *sylvestris/nigra* (8) | 19; 34 |
| **ONDARO (Basque Country, Spain)** | Gravettian | 396 | *Juniperus* sp. (363), *Quercus* subg. *Quercus* (15), cf. *Rosacea* (18) | 88 |
| **TÊTE DU LION (Ardèche, France)** | Final Gravettian-Solutrean | 1 | *Pinus sylvestris* (1) | 89; 90 |
