# Supplementary material for: The conquest of the dark spaces: An experimental approach to lighting systems in Paleolithic caves
Source: PLoS One. 2021 Jun 16;16(6):e0250497. doi: 10.1371/journal.pone.0250497 (PMC8208548; doi:10.1371/journal.pone.0250497)
Supplement: S2 Appendix — (PDF) [file pone.0250497.s002.pdf]

***S2 Appendix*: Experimental cave data**

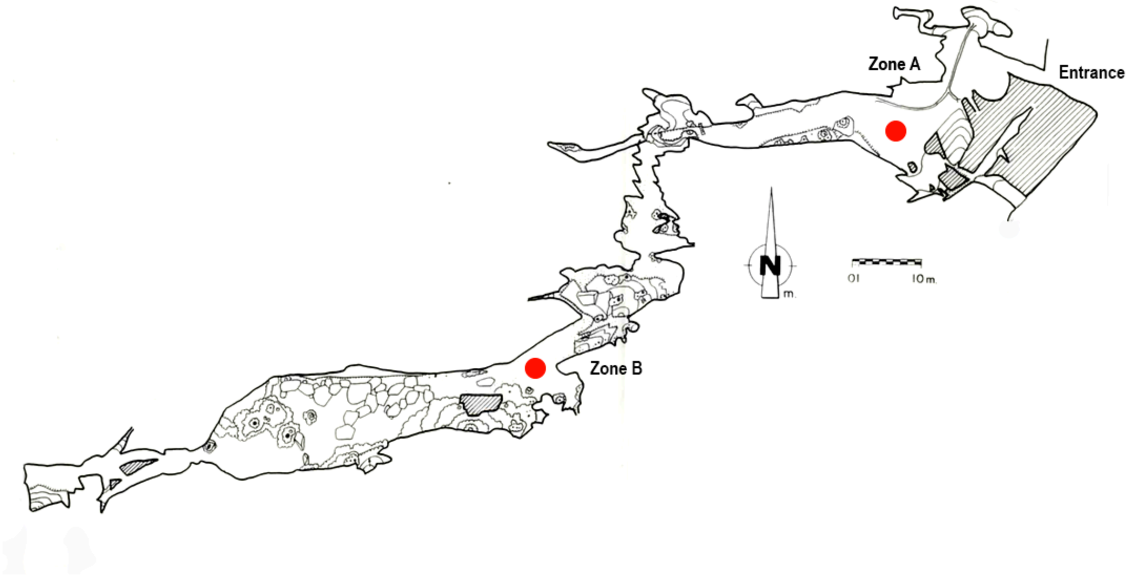

Planimetry of the "experimental" cave where we point out the chambers where we have developed the experiments (modified from AYALA *et al*. 1986: 167).
