# Supplementary material for: The conquest of the dark spaces: An experimental approach to lighting systems in Paleolithic caves
Source: PLoS One. 2021 Jun 16;16(6):e0250497. doi: 10.1371/journal.pone.0250497 (PMC8208548; doi:10.1371/journal.pone.0250497)
Supplement: S3 Appendix — (PDF) [file pone.0250497.s003.pdf]

***S3 Appendix*: Measured tools data**

The lux meter used in the experiments was a model PCE-L335. Its technical specifications are as follows (information is taken from pce-iberica.es) (Table 1):

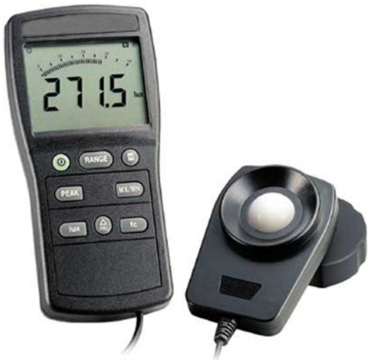

**Ranges**	40/400/4.000/40.000/400.000 lux<br>240/400/4.000/40.000 foot-candle
**Resolution**	0.01 lux<br>0.1 foot candle
**Accuracy**	± 3 %
**Overcome indicator**	OL = Overload
**CIE photopic f`1**	< 9 %
**Light sensor**	silicon photodiode
**Temperature characteristic**	± 0,1 %/°C
**Ambient condition**	0...50°C/ < 80 % H.r.
**Screen**	LCD 3½ positions
**Alimentation**	Six battery micro AAA
**Dimensions**	Light sensor: 100 x 60 x 27 mm<br>Apparatus: 135 x 72 x 33 mm
**Weight**	250 g
**Regulation**	CNS 5199 II

Table 1. Specification techniques of PCE-L335 lux meter (information is taken from pce-iberica.es)

The infrared thermometer used to measure the temperature was a model PCE-778. Its technical specifications are as follows (PCE Instruments website) (Table 2).

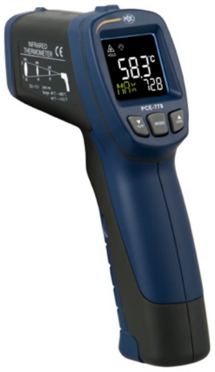

**Optical resolution**	12: 1
**Measuring range**	-40... 800°C/-40... 1472°F
**Measurement accuracy**	Measuring range -40... 0°C/-40... 32°F: ± 4°C/7.2°F<br>Measuring range 0... 400°C/32... 752°F: ± 1.5°C/2.7°F<br>Measuring range 400... 800°C/752... 1472°F: ± 2°C/3.6°F
**Resolution**	0.1°C/0.18°F at 0... 199.9°C/32... 391.8°F<br>1°C/1.8°F at > 199.9°C/391.8°F
**Emissivity**	0.1... 1 (adjustable)
**Spectral**	8... 14 µm
**Polarity**	No indication: positive polarity<br>Display "-": negative polarity
**Automatic shutdown**	After 30 seconds of inactivity
**Operating conditions**	0... 50°C/32... 122°F, rH: 10... 90 %
**Storage conditions**	-20... 60°C/-4... 140°F, rH <80 %
**Power supply**	9 V block battery
**Laser**	2nd grade
**Weight**	250 g/< 1 lb
**Dimensions**	95 x 83 x 192 mm/3.7 x 3.3 x 7.6 in

Table 2: Specification techniques of the PCE-778 infrared thermometer (taken from pce-instruments.com)
