# Supplementary material for: The conquest of the dark spaces: An experimental approach to lighting systems in Paleolithic caves
Source: PLoS One. 2021 Jun 16;16(6):e0250497. doi: 10.1371/journal.pone.0250497 (PMC8208548; doi:10.1371/journal.pone.0250497)

***S4 Appendix*: Experimental data**

The technical data sheet includes the experiment's location, date, raw materials used, manufacturing, duration, and photography of the lighting system, as well as the lighting data (illuminance, light intensity, and radius) and temperature. In some experiments, we included the illuminance value before and after the relight and continuous monitoring of the illuminance (lux) emitted in relation to the measured distance from the center of the flame.

4.1. Experimental torches
4.2. Experimental lamps
4.3. Experimental fireplace

4.1. Experimental torches

EXPERIMENT 1

- **Experimentation site:** Isuntza I cave (inner context, Zone A).
- **Date:** 23-10-2017
- **Raw materials:** juniper woods in a dry state (733 g), dry birch bark (30 g), and ivy in the green state (50 g). The drying time of the wood was 41 days in a sheltered and humidity-free place.
- **Manufacturing:** central core of juniper branch 2 cm. diameter and crushed, to give it greater fibrosity and facilitate oxygenation. Around it, we added 30 grams of birch bark and 5 fragments of juniper heartwood cut tangentially, approximately 4 cm. wide and 0.5 cm. thick. All this was tied to the central shaft with ivy. Additionally, we added an oak handle 5 cm. thick and 10 cm. long (this did not affect combustion).
- **Weight:** 813 g.
- **Length:** 47 cm.
- **Width:** 8 cm.
- **Duration:** 31´.

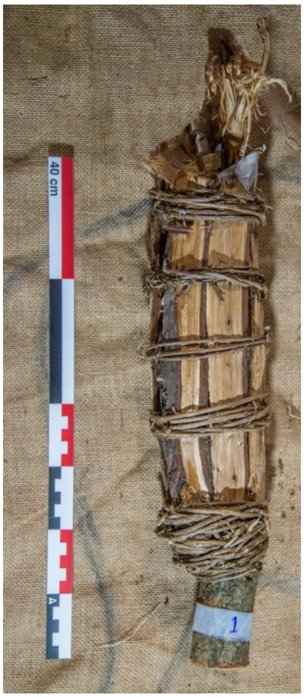

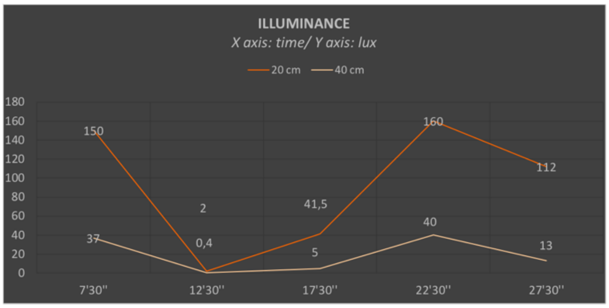

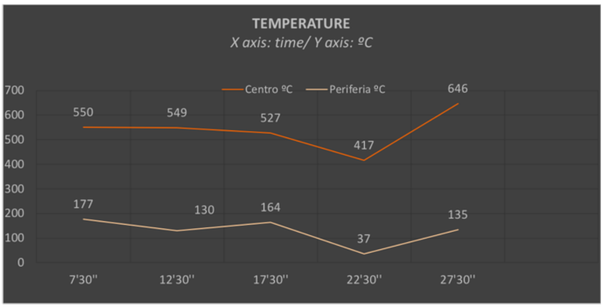

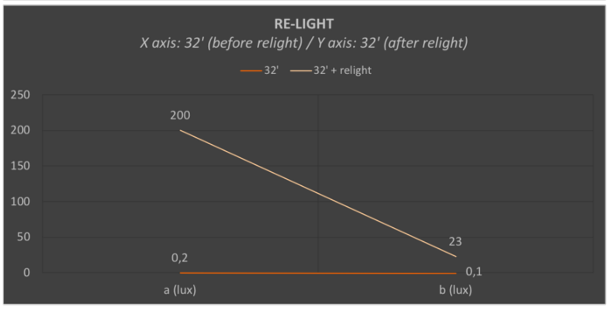

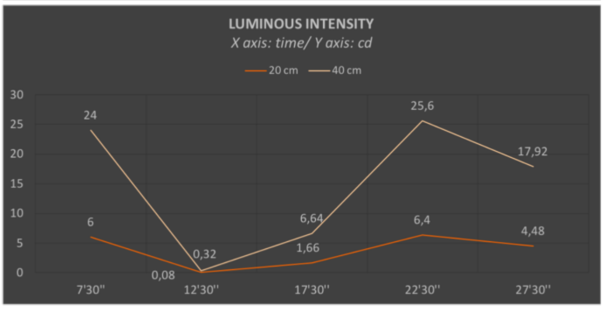

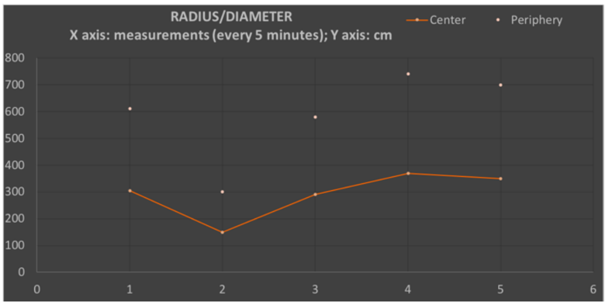

EXPERIMENT 2:

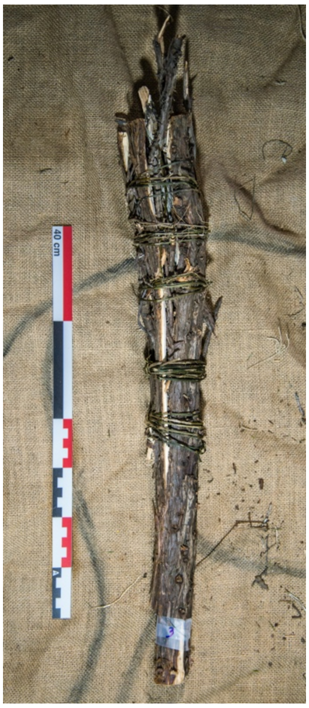

- **Experimentation site:** Isuntza I cave (inner context, Zone A).

- **Date:** 23-10-2017

- **Raw materials:** juniper woods in a dry state (338 g), dry birch bark (15 g), and ivy in the green state (25 g). The drying time of the wood was 41 days in a sheltered and humidity-free place. Additionally, we added deer marrow (30 g) and Pinus pinea resin (solid-state, 30 g, various fragments of 1-3 cm.) in this experiment.

- **Manufacturing:** juniper branch of 5 cm. thick, crushed, with 7 longitudinal fractures at the top and middle, except at its proximal end, which will serve as a handle. The interior was filled with 15 grams of birch bark and 30 grams of *Pinus pinea* resin. Five ivy ribbons were used to give it greater stability. These were tied around the tool. Finally, its exterior part was impregnated with 30 grams of fresh deer marrow.

- **Weight:** 438 g.

- **Length:** 57 cm.

- **Width:** 6 cm.

- **Duration:** 21´.

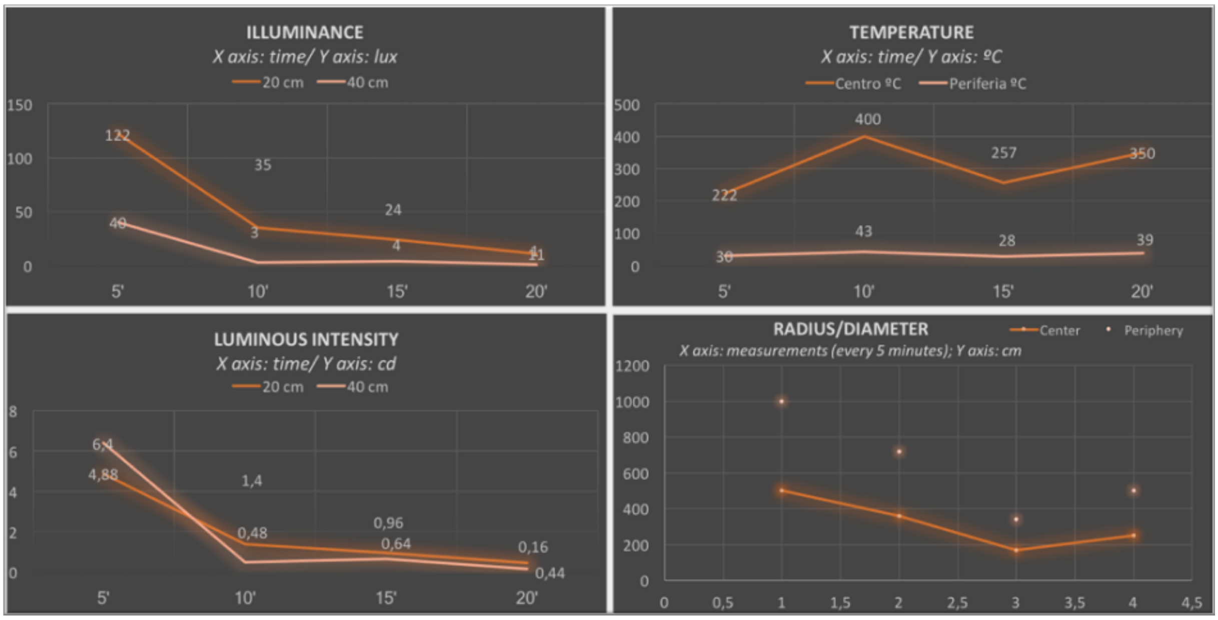

Experiment 3:

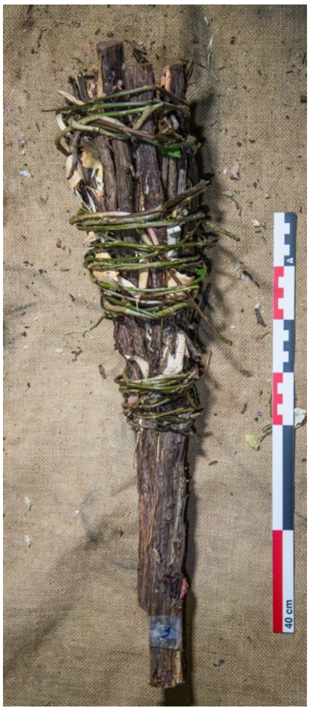

- **Experimentation site:** Isuntza I cave (inner context, Zone A).
- **Date:** 23-10-2017
- **Raw materials:** juniper woods in a dry state (742 g), dry birch bark (30 g), and ivy in the green state (50 g). The drying time of the wood was 41 days in a sheltered and humidity-free place. Additionally, we added deer marrow (60 g) and *Pinus pinea* resin (30 g, various fragments of 1-3 cm.).
- **Manufacturing:** The bone marrow was spread on these four axes. The interior has filled with birch bark and resin. Unlike the previous experiment, 5 juniper branches of 1-2 cm. of diameter have been added to the interior. Thick as additional woody fuel. Additionally, 5 ivy ribbons were used to give it greater stability. These were tied around the tool.
- **Weight:** 912 g.
- **Length:** 55 cm.
- **Width:** 11 cm.
- **Duration:** 44'.´

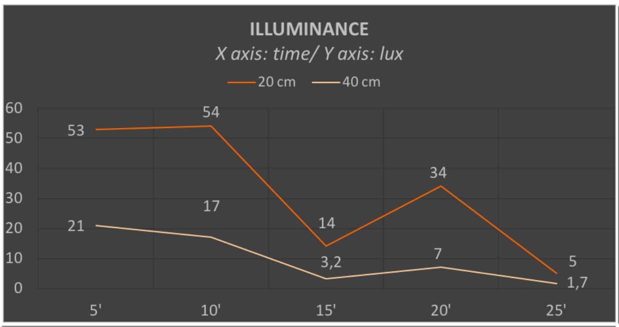

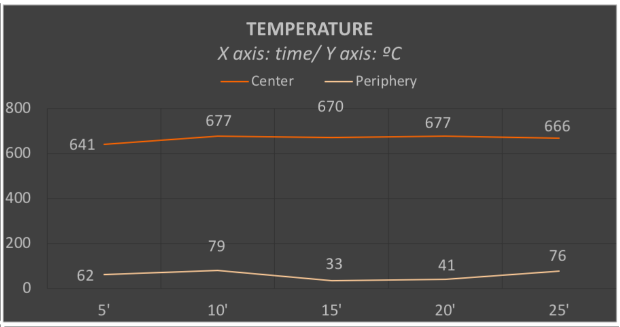

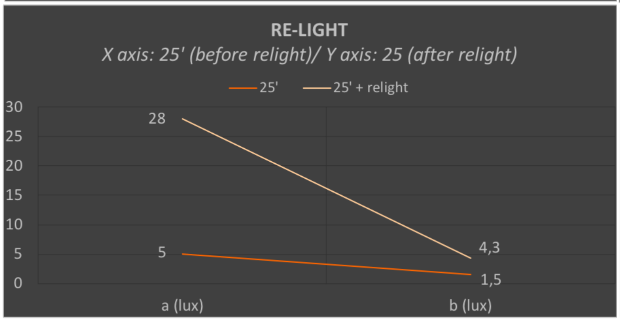

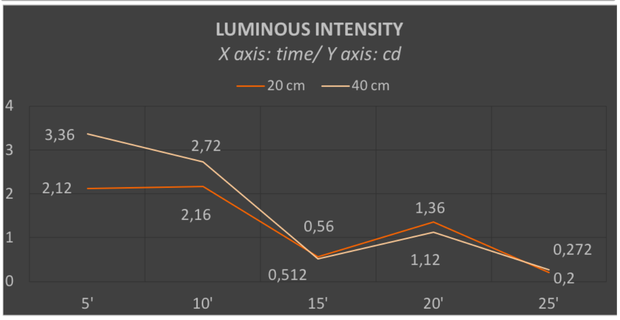

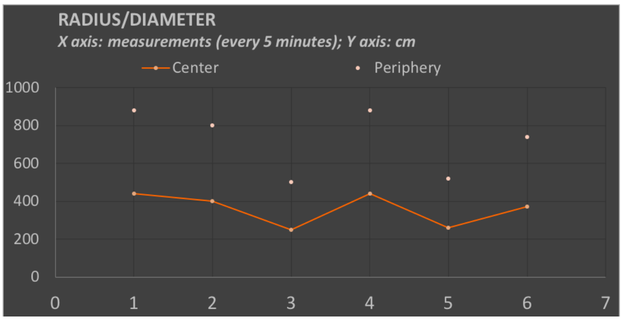

EXPERIMENT 4:

**** Experiment similar to 3, only the wood had 43 more days of drying.***

- **Experimentation site:** Isuntza I cave (inner context, Zone B).

- **Date:** 17-2-2018

- **Raw materials:** juniper woods in a dry state (742 g), dry birch bark (30 g), and ivy in the green state (50 g). The drying time of the wood was 124 days in a sheltered and humidity-free place. Additionally, we added deer marrow (60 g) and *Pinus pinea* resin (30 g, solid-state, various fragments of 1-3 cm.).

- **Manufacturing:** *Experiment similar to 3 (see adobe)*

- **Weight:** 912 g.

- **Length:** 55 cm.

- **Width:** 11 cm.

- **Duration:** 50´

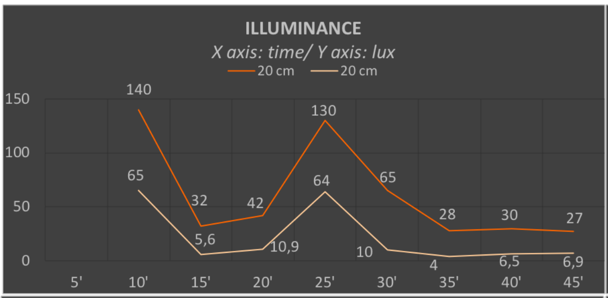

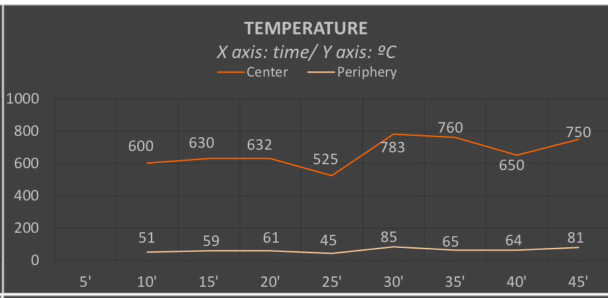

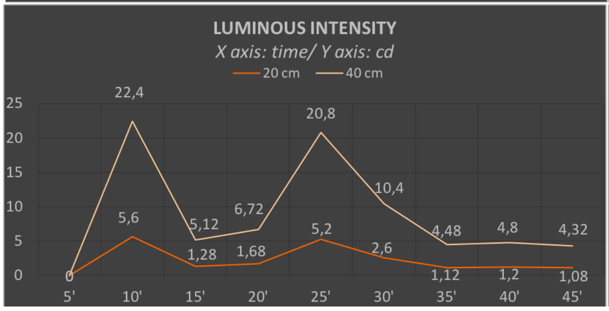

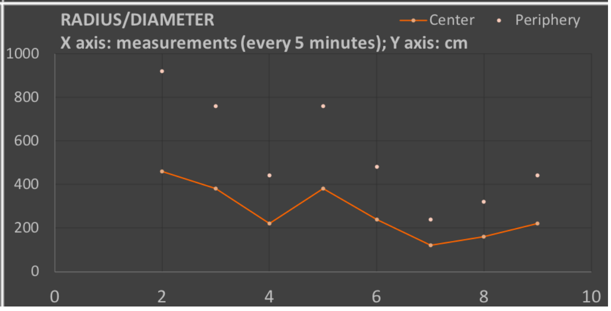

Experiment 5:

**** Experiment similar to 3 and 4, only the wood had 88 more days of drying (than experiment 3), and no resin was added, just bone marrow. The weight of the resin (30 g.) was exchanged with a higher quantity of wood.***

- **Experimentation site:** Isuntza I cave (inner context, Zone B).
- **Date:** 17-2-2018
- **Raw materials:** juniper woods in a dry state (772 g), dry birch bark (30 g), and ivy in the green state (50 g).
  The drying time of the wood was 215 days in a sheltered and humidity-free place. Additionally, we added deer marrow (60 g).
- **Manufacturing:** *Experiment similar to 3 and 4 (see adobe) but without resin.*
- **Weight:** 912 g.
- **Length:** 55 cm.
- **Width:** 11 cm.
- **Duration:** 61´.

Min./cm.	10	20	30	40	50	60	70	80	90	100	110	120	130	140	150	160	170	180	190	200	210	220	230
5'	34	3	3	5	2,4	1,3	6	2,7	1,5	1	0,9	1	0,6	0,3	0,2	0	0	0	0	0	0	0	0
10'	120	83	36	14	8	7	6,1	4	2,8	1,8	1,3	1,2	0,8	0,5	0,1	0	0	0	0	0	0	0	0
15'	300	262	123	92	88	64	40	29	21	17	14	10	5,7	5,3	3,9	3,3	3	2,8	2,7	2,6	2,1	1,8	1,7
20'	148	85	46	27	22	13	14	10	7	6	4,8	4,3	3,4	2,5	2	1,9	1,7	1,3	1	1	0,9	0,7	0,6
25'	84	40	9	3,6	3,7	3,7	3,2	3	2,7	2,2	0,9	0,7	0,6	0,4	0,2	0,2	0,2	0,3	0,1	0,1	0,1	0,1	0
30'	108	46	24	16	10,7	8,6	6,5	4	3,3	2	1,9	1,8	1,6	1,5	1,3	1,1	1	0,9	0,8	0,4	0,3	0,3	0,3
35'	12	7	3	2	1,9	1,8	0,8	1	1,1	1,3	1,3	0,6	0,5	0,4	0,2	0,2	0,2	0,1	0,1	0,1	0,1	0,1	0
40'	48	30	13	6,9	5	4	2,3	1,2	0,8	0,5	0,4	0,4	0,2	0,2	0,1	0,1	0	0	0	0	0	0	0
45'	91	66	49	31	20,8	9,9	7,9	4,7	3,9	2,3	1,7	1,5	1,3	1	0,9	0,6	0,5	0,4	0,3	0,2	0,2	0,1	0,1

240	250	260	270	280	290	300	310	320	330	340	350	360	370	380	390	400	410	420	430
0	0	0	0	0	0	0	0	0	0	0	0	0	0	0	0	0	0	0	0
0	0	0	0	0	0	0	0	0	0	0	0	0	0	0	0	0	0	0	0
1,2	1,2	1	0,9	0,8	0,7	0,7	0,6	0,5	0,5	0,4	0,3	0,3	0,2	0,2	0,2	0,2	0,2	0,2	0,1
0,5	0,4	0,4	0,3	0,2	0,3	0,4	0,4	0	0	0	0	0	0	0	0	0	0	0	0
0	0	0	0	0	0	0	0	0	0	0	0	0	0	0	0	0	0	0	0
0,2	0,2	0,1	0,1	0,1	0,1	0	0	0	0	0	0	0	0	0	0	0	0	0	0
0	0	0	0	0	0	0	0	0	0	0	0	0	0	0	0	0	0	0	0
0	0	0	0	0	0	0	0	0	0	0	0	0	0	0	0	0	0	0	0
0	0	0	0	0	0	0	0	0	0	0	0	0	0	0	0	0	0	0	0

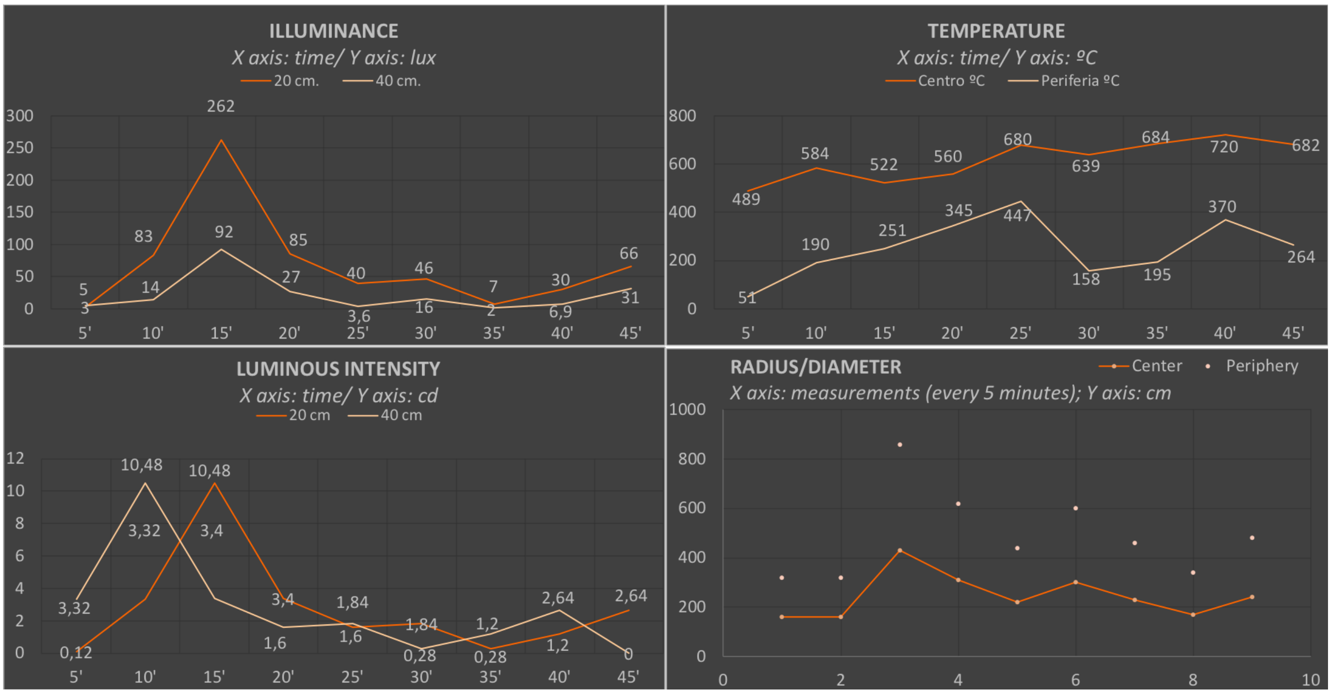

4.2. Experimental lamps

**** The lamp employed in the experiments was a replica of the one located in the La Mouthe Cave (Rivière 1899, 1901) and similar to the lamp located in Bisons Hall of Atxurra cave (Medina-Alcaide 2019, Garate et al. 2020). Its dimensions are 17 cm. high, 12 cm. wide and includes a cavity with ≈150 cm³ of deposit.***

Experiment 6:

- **<u>Experimental site:</u>** Isuntza I cave (inner context, Zone A).
- **<u>Data:</u>** 16-05-2018
- **<u>Raw materials:</u>** two juniper branches in a dry state (20 g) of 3 by 0.5 cm.. The drying time of the wood was 215 days in a sheltered and humidity-free place. Additionally, we added bovid marrow (23 g).
- **<u>Manufacturing:</u>** We fill the lamp's cavity with the bone marrow and place the juniper branches in the center, crushed to favor the absorption action of the fat fuel. Then, we position them pyramid-shaped, supported by their upper ends in the cavity center.
- **<u>Duration:</u> >60'.**

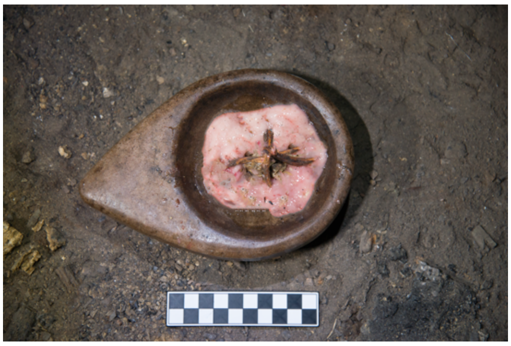

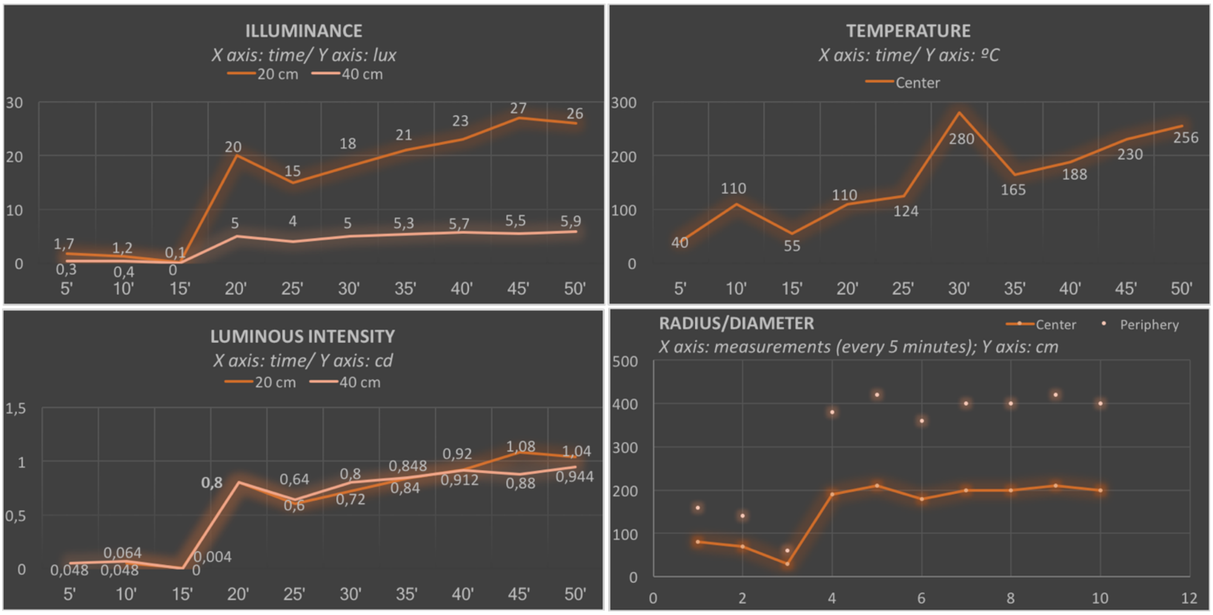

Min./cm. (lux)	10	20	30	40	50	60	70	80	90	100	110	120	130	140	150	160	170	180	190	200	210
5'	7	1,7	0,8	0,3	0,3	0,2	0,1	0													
10'	3,8	1,2	0,8	0,4	0,2	0,1	0														
15'	0,4	0,1	0																		
20'	75	20	9,5	5	3,3	2,3	1,9	1,3	7	0,8	0,6	0,5	0,4	0,3	0,3	0,2	0,2	0,1	0,1	0	
25'	55	15	7	4	3	1,8	1,4	1,2	0,8	0,6	0,5	0,4	0,4	0,3	0,3	0,2	0,2	0,1	0,1	0,1	0
30'	39	18	7,9	5	3	1,9	1,5	1,2	0,8	0,6	0,5	0,4	0,4	0,3	0,2	0,1	0,1	0			
35'	91	21	9,9	5,3	3,2	2,1	1,4	1	0,8	0,7	0,5	0,4	0,3	0,3	0,2	0,2	0,2	0,1	0,1	0	
40'	109	23	9,4	5,7	3,1	2,1	1,5	1,1	1	0,7	0,6	0,5	0,4	0,3	0,3	0,3	0,2	0,2	0,1	0	
45'	85	27	12	5,5	3,2	2,4	1,6	1,2	1	0,8	0,7	0,5	0,4	0,4	0,2	0,2	0,1	0,1	0,1	0	
50'	90	26	11	5,9	3,3	2,4	1,9	1,1	0,9	0,7	0,6	0,4	0,3	0,3	0,3	0,2	0,2	0,1	0,1	0,1	0

Experiment 7:

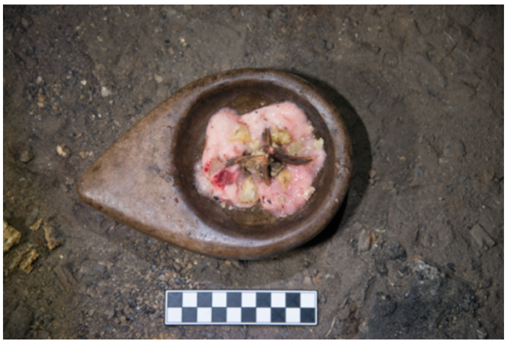

- **Experimental site:** Isuntza I cave (inner context, Zone A).
- **Data:** 23-10-2017
- **Raw materials:** two juniper branches in a dry state (20 g) of 3 by 0.5 cm. The drying time of the wood was 215 days in a sheltered and humidity-free place. Additionally, we added bovid marrow (23 g) and *Pinus pinea* resin (12 g, solid-state, various fragments of 0.5-1 cm.).
- **Manufacturing:** We fill the lamp's cavity with the bone marrow and place the juniper branches in the center, crushed to favor the absorption action of the fat fuel. Then, we position them pyramid-shaped, supported by their upper ends in the cavity center. The resin fragments were placed in the fat (scattered) and around the wick.
- **Duration: >60'.**

Min./cm.(lux)	10	20	30	40	50	60	70	80	90	100	110	120	130	140	150	160	170	180	190	200
5'	82	10,3	6,5	5,3	3,6	2,9	2,2	1,2	1,1	0,9	1,5	0,7	0,5	0,4	0,4	0,2	0,2	0,2	0	0
12'	107	15	9,7	4,7	3,5	2	1,4	1	0,8	0,6	1	0,5	0,4	0,3	0,2	0,1	0	0	0	0
25'	67	15	8,1	3,8	2,4	1,7	1,3	1,7	0,5	0,4	1	0,3	0,2	0,2	0,1	0,1	0,1	0	0	0
30'	73	12	6,2	3,5	2	1,2	0,7	0,5	0,4	0,4	0,6	0,3	0,3	0,2	0,2	0,1	0,1	0,1	0	0
35'	52	3,2	3	2	1,3	0,6	0,3	0	0	0	0,2	0	0	0	0	0,2	0,1	0,1	0,1	0
40'	61	53	2	1,5	0,6	0,4	0,3	0,1	0	0	0,1	0	0	0	0	0	0	0	0	0
45'	64	8,8	4,8	2,2	1,3	0,8	0,4	0,2	0,2	0,1	0,3	0,1	0	0	0	0	0	0	0	0
50'	112	13	6,3	4,5	3,1	1,7	1,6	0,9	0,7	0,5	1,2	0,4	0,3	0,2	0,2	0	0	0	0	0
55'	91	11,1	5,2	2,4	1,2	1,3	0,7	0,2	0,2	0,1	0,4	0,1	0	0	0	0	0	0	0	0
60'	67	8,1	4,2	2,3	1,6	1	0,7	0,3	0,2	0,1	0,4	0	0	0	0	0	0	0	0	0
65'	8,3	9,5	2,5	1,7	1	0,7	0,5	0,2	0,1	0	0,3	0	0	0	0	0	0	0	0	0
70'	54	9,7	3,5	1,7	0,7	0,6	0,3	0	0	0	0,1	0	0	0	0	0	0	0	0	0

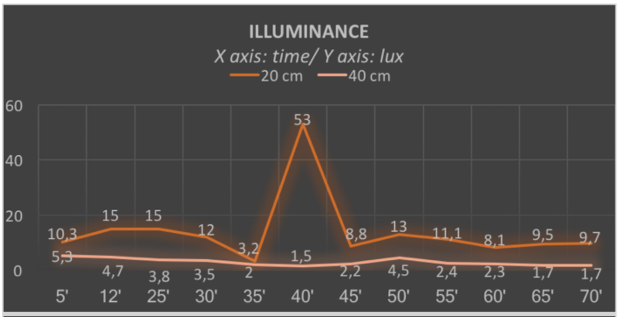

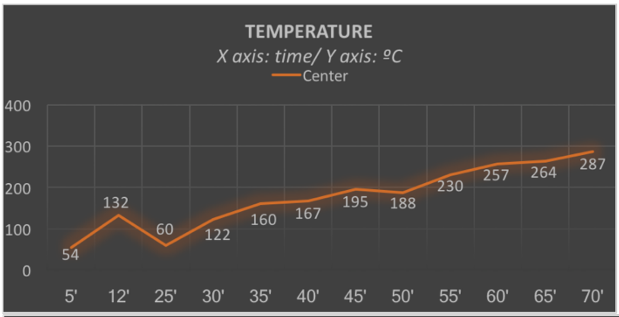

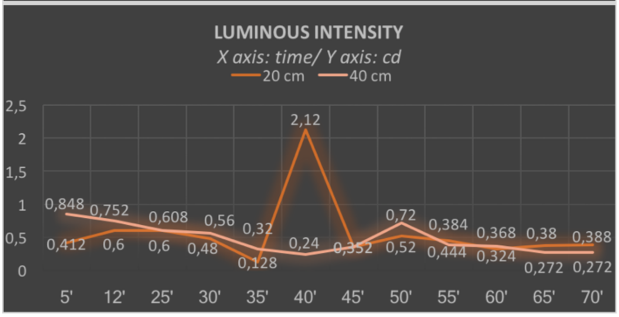

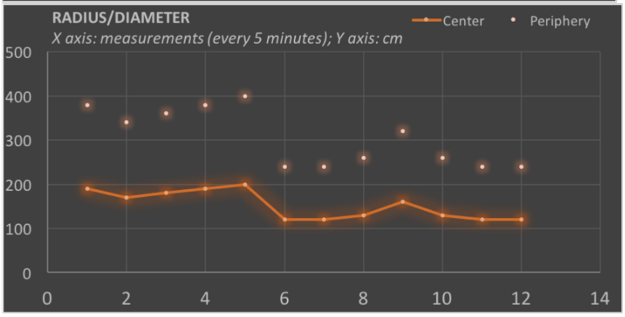

4. 3. Experimental fireplace

Experiment 8:

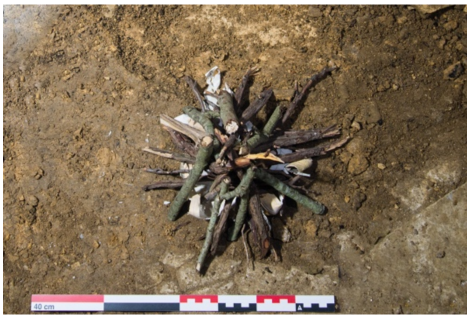

- **<u>Experimental site:</u>** Isuntza I cave (inner context, Zone A).
- **<u>Data:</u>** 23-10-2017
- **<u>Raw materials:</u>** Five hundred g. of branches of 1-2 cm. of juniper and 400 g. of branches of 2-2.5 cm. of oak in a dry state. The wood was in a dry state (41 days of drying). We also add 12 g. of birch bark to enhance the start of the flame.
- **<u>Diameter:</u>** 23 cm.
- **<u>Height:</u>** 7 cm. (tepee shape).

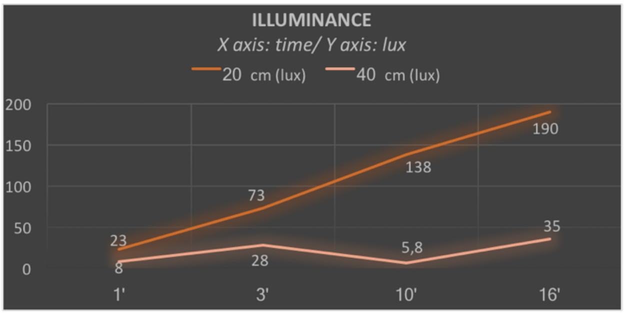

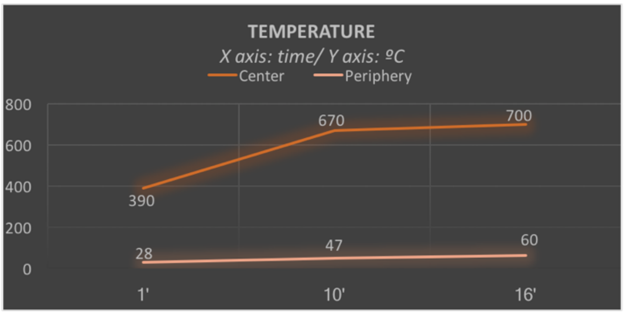

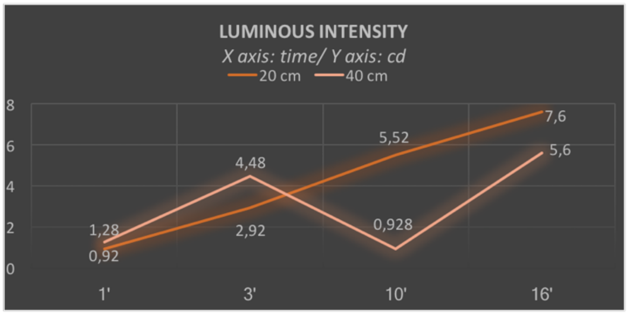

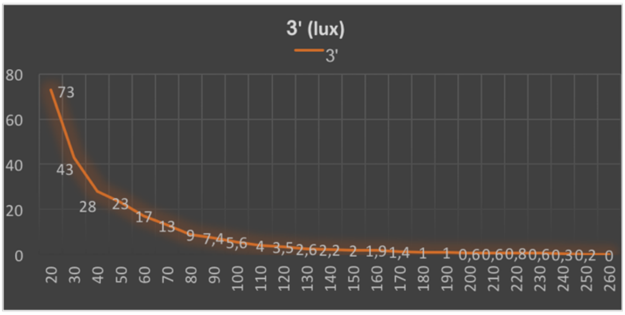

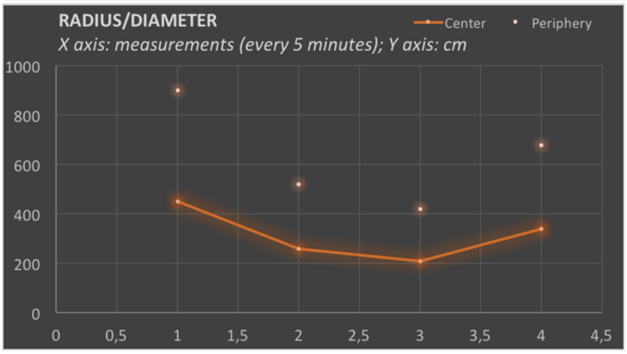

Supplement: S4 Appendix — (PDF) [file pone.0250497.s004.pdf]
