# Supplementary material for: The conquest of the dark spaces: An experimental approach to lighting systems in Paleolithic caves
Source: PLoS One. 2021 Jun 16;16(6):e0250497. doi: 10.1371/journal.pone.0250497 (PMC8208548; doi:10.1371/journal.pone.0250497)
Supplement: S5 Appendix — An idea. (PDF) [file pone.0250497.s005.pdf]

S5 Appendix: torch-lamp model. An idea.

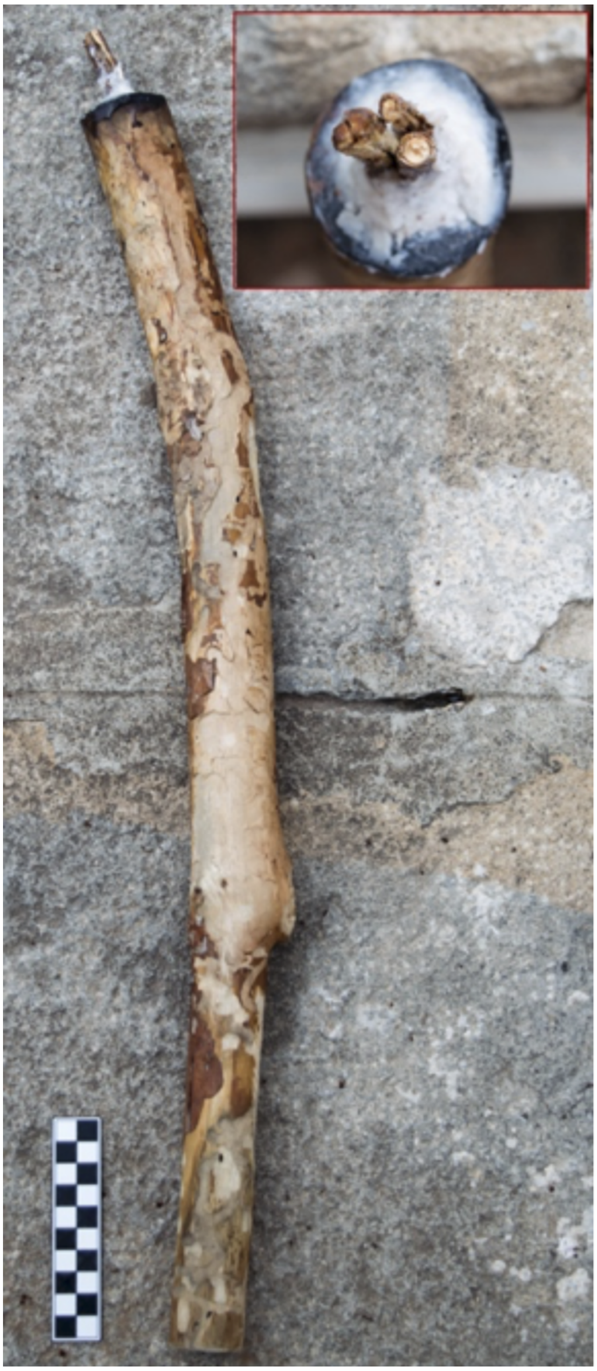

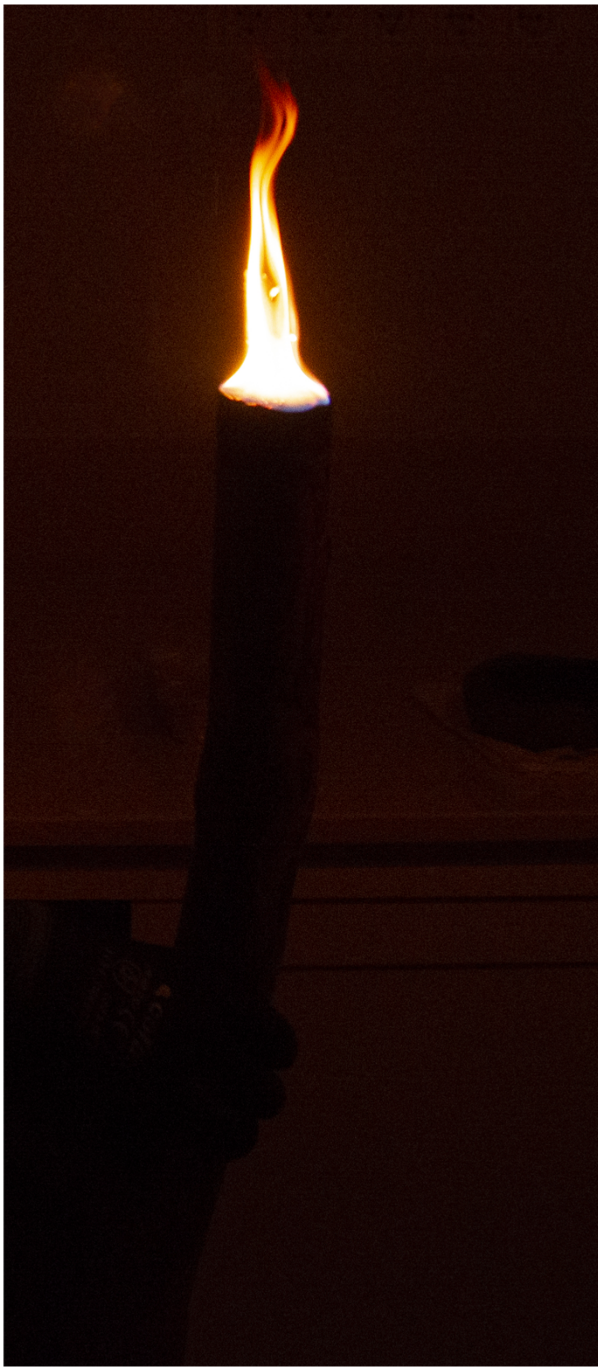

Observation:

This torch lamp model has been made on a 4 to 6 cm. thick wooden branch (another elongated perishable support, for example, bone, could also serve as support). At the top of the branch, a concavity 10 cm. deep and 3-4 cm. in diameter was opened. Animal fat and wicks (three thin wooden branches) were inserted. The latter would produce the flame once the fat has been melted through absorption by capillary action. To obtain intense light, it is necessary to include several wicks (or add resin on time), and the wicks should protrude as much as possible from the hollow without burning.

This tool is comfortable to transport if we do not overfill the cavity with fatty fuel, thus avoiding fuel spills. Remember that for an hour of operation, only 40 g of fatty fuel is required (Beaune 1987).

The archaeological record left by using this mixed lighting system was practically null since it is made of perishable material (which, except for the upper flange of the branch, does not char). We have verified that only a few small charcoals from the wick are spilled when it is lit because the wick partially burns until it begins to absorb fat. The use of this tool in the underground environment could be difficult to determine unless it was set on the ground in such a way as to produce a hole that could be preserved in the clay. In this sense, we must remember that the absence of evidence of wooden instruments inside the caves (linked to the exploration and underground stay) does not mean the evidence of absence but is conditioned on the wood's difficult preservation without carbonization.

- **References-supporting information.**

78. Romano M, Citton P, Salvador I, Arobba D, Rellini I, Firpo M et al. A multidisciplinary approach to a unique Palaeolithic human ichnological record from Italy (Bàsura Cave). eLife 2019;8: e45204. doi: 10.7554/eLife.45204.
79. Quiles A, Valladas H, Bocherens H, Delqué-Količ E, Kaltnecker E, van der Plicht J, et al. First high-precision chronological model for the decorated Upper Paleolithic cave of Chauvet-Pont d'Arc, Ardèche, France. Proceedings of the National Academy of Sciences of the USA 2016;113(17): 4670-4675.
80. Casabó J, de Dios-Boronat J, Carrión Y, Esquembre MA, Guillem PM, Martínez-Valle R, et al. New evidence of Paleolithic rock art at the Cova del Comte (Pedreguer, Spain): Results of the first surveys. Quat. Int. 2016;432: 25-40.
81. Clottes J, Courtin J, Vanrell L. Cosquer redécouvert. Paris: Seuil; 2005.
82. Valladas H, Quiles A, Delque-Kolic M, Kaltnecker E, Moreau C, Pons-Branchu E, et al. Radiocarbon dating of the decorated Cosquer cave (France). Radiocarbon 2017;59(2): 621-633.
83. Jaubert J, Genty D, Valladas H, Camus H, Courtaud P, Ferrier C, et al. The chronology of human and animal presence in the decorated and sepulchral cave of Cussac (France). Quat. Int. 2016;432: 5-24.
84. Garate D, Bourrillon R, Rios-Garaizar J. La grotte ornée paléolithique d'Etxeberri (Camou- Cihige Pyrénées-Atlantiques): datation du contexte archéologique de la salle des Peintures. BSPF 2012;109(4): 637-650.
85. Arias P. Rites in the dark? An evaluation of the current evidence for ritual areas at Magdalenian cave sites. World Archaeol. 2009;41(2): 262-294.
86. Medina-Alcaide MA. Los restos vegetales carbonizados del contexto arqueológico interno de Morgota koba (Kortezubi Bizkaia). Kobie 2015;5: 105-119.
87. Pigeaud R, Primault J. Une première date pour la grotte ornée du Moulin de Laguenay (Lissac-sur- Couze, Corrèze). Préhistoire du Sud-Ouest 2007;14(1): 161-162.
88. Ruiz-Alonso M. Maderas carbonizadas en la cueva de Ondaro (Nabarniz, Bizkaia). Kobie, Paleoantropol. 2018;35: 57-66.
89. Combier J. La grotte de la Tête-du-Lion à Bidon (Ardèche). In: Leroi-Gourhan A, editor. L'Art des cavernes. Atlas des grottes ornées paléolithiques françaises. Paris: Imprimerie Nationale; 1984. pp. 595-599.
90. Monney J, Combier J, Kaltnecker E, Lateur N, Moreau C, Stocchetti S, et al. Nouveaux éléments de discussion chronologiques dans le paysage des Grottes ornées de l'Ardèche: Oulen, Chabot et Tête-du-Lion. In: Paillet P, editor. Les arts de la Préhistoire: micro-analyses, mises en contextes et conservation. Paris: Paléo numéro special; 2014. pp. 271-283
